# Targeting NK-1R attenuates renal fibrosis *via* modulating inflammatory responses and cell fate in chronic kidney disease

**DOI:** 10.3389/fimmu.2023.1142240

**Published:** 2023-03-24

**Authors:** Enyi Zhu, Yang Liu, Ming Zhong, Yu Liu, Xi Jiang, Xiaorong Shu, Na Li, Hui Guan, Yin Xia, Jinhong Li, Hui-yao Lan, Zhihua Zheng

**Affiliations:** ^1^ Department of Nephrology, Center of Kidney and Urology, the Seventh Affiliated Hospital, Sun Yat-sen University, Shenzhen, China; ^2^ Department of Clinical Laboratory, the Seventh Affiliated Hospital, Sun Yat-sen University, Shenzhen, China; ^3^ Department of Cardiology, Sun Yat-sen Memorial Hospital of Sun Yat-sen University, Guangzhou, China; ^4^ Faculty of Medicine, School of Biomedical Sciences, The Chinese University of Hong Kong, Hong Kong, Hong Kong SAR, China; ^5^ Departments of Medicine & Therapeutics, Li Ka Shing Institute of Health Sciences, Lui Che Woo Institute of Innovative Medicine, The Chinese University of Hong Kong, Hong Kong, Hong Kong SAR, China; ^6^ Guangdong-Hong Kong Joint Laboratory for Immune and Genetic Kidney Disease, Guangdong Provincial People’s Hospital and Guangdong Academy of Medical Sciences, Guangzhou, China

**Keywords:** targeted therapy, neuropeptide, substance P, NK-1R, renal inflammation, macrophages, renal fibrosis, chronic kidney disease

## Abstract

**Background:**

Renal fibrosis is the final common pathway of chronic kidney disease (CKD), which is clinically irreversible and without effective therapy. Renal tubules are vulnerable to various insults, and tubular injury is involving in the initiation and evolution of renal inflammation and fibrosis. Neurokinin-1 receptor (NK-1R) functions by interacting with proinflammatory neuropeptide substance P (SP), exerting crucial roles in various neurological and non-neurological diseases. However, its roles in renal inflammation and fibrosis are still unknown.

**Methods:**

We collected renal biopsy specimens and serum samples of individuals with or without CKD. Additionally, knockout mice lacking NK-1R expression, SP addition and NK-1R pharmacological antagonist treatment in the unilateral ureteral obstruction (UUO) model, and NK-1R-overexpressed HK-2 cells were employed.

**Results:**

Renal SP/NK-1R and serum SP were increased in patients with CKD and mice experiencing UUO and correlated with renal fibrosis and function. SP addition enhanced UUO-induced progressive inflammatory responses and renal fibrosis, whereas genetically or pharmacologically targeting NK-1R attenuated these effects. Mechanistically, TFAP4 promoted NK-1R transcription by binding to its promoter, which was abolished by mutation of the binding site between TFAP4 and NK-1R promoter. Furthermore, SP acted through the NK-1R to activate the JNK/p38 pathways to modulate cell fate of tubular epithelial cells including growth arrest, apoptosis, and expression of profibrogenic genes.

**Conclusion:**

Our data reveals that SP/NK-1R signaling promotes renal inflammatory responses and fibrosis, suggesting NK-1R could be a potential therapeutic target for the patients with CKD.

## Introduction

Renal fibrosis, regarded as the key pathological process leading to the chronic kidney disease (CKD), is becoming a grievous public-health concern (1, 2). Renal tubules are the dominant element of the kidney, and increasing research is focused on the causative effect of renal tubular epithelial cells (renal TECs) on the initiation and evolution of renal fibrosis (3). Upon renal injuries, TECs undergo various abnormal changes, including cell cycle arrest, apoptosis, and cellular senescence as well as partial epithelial-to-mesenchymal transition (EMT), which consequently facilitates renal fibrosis by generating proinflammatory and profibrogenic responses *via* autocrine and paracrine effects (4, 5). Accumulating evidence shows that kidney fibrogenesis involves the collaboration of multiple signaling pathways, such as transforming growth factor-β (TGF-β)/SMAD, mitogen-activated protein kinases (MAPK), nuclear factor-κB (NF-κB), and Wnt/β-catenin (6–9). Nevertheless, the fundamental mechanisms that drives renal TECs to trigger renal tubulointerstitial fibrosis remain to be determined.

Neurokinin-1 receptor (NK-1R) is encoded by the *TACR1* gene and serves as the high-affinity G protein-coupled receptor for substance P (SP), which is encoded in exon 3 of the *TAC1* gene and represents a proinflammatory neuropeptide of tachykinin family. NK-1R is widely detected in multiple cell types, including nerve cells (e.g., microglia, neurons, and astrocytes), immune cells (e.g., dendritic cells, lymphocytes, and macrophages), endothelial cells, smooth muscle cells, together with epithelial cells (10, 11). SP is also extensively distributed throughout not only in neuronal but also in non-neuronal tissues as well as in the body fluids including blood (12). SP/NK-1R axis exerts critical effects on multifarious cell activities, such as apoptosis, chemotaxis, and inflammation, and thus participates in regulating various physiological/pathological processes, including pain, emesis, neurological diseases (e.g., pruritus, epilepsy, Alzheimer’s disease), cardiovascular diseases (e.g., heart failure, cardiomyopathy, and myocardial infarction), inflammatory diseases (e.g., rheumatoid arthritis, osteoarthritis, and psoriasis), and cancer (13).

NK-1R antagonists are presently being studied as analgesic, anti-emetic, and anti-inflammatory drugs, which have been approved for preventing chemotherapy-induced nausea and emesis by the U.S. Food and Drug Administration (14). Previous studies have revealed that SP/NK-1R may play a critical role in fibrotic disorders, such as myocardial fibrosis and liver fibrosis (15). During aortocaval fistula-mediated volume overload, the SP/NK-1R axis promotes adverse myocardial remodeling by activating cardiac mast cells, resulting in elevated levels of tumor necrosis factor-α (TNF-α) together with matrix metalloproteinase (MMP) activity, and later ECM remodeling (16). In cytokine-mediated liver injury, inactivation of SP/NK-1R significantly reduces inflammatory cell infiltration and liver cell apoptosis (17). Moreover, upon cholestatic liver injury, serum SP level and SP/NK-1R expression in the liver are up-regulated, leading to SP/NK-1R pathway hyperactivation, which promotes liver fibrosis by mediating cell aging in hepatic stellate cells and cholangiocytes as well as enhancing the biliary secretion of TGF-β1 (18). However, the impact of the SP/NK-1R pathway on TEC dysfunction and consequent renal fibrosis is still unclear.

The present work revealed highly increased SP/NK-1R levels within the kidneys and serum SP levels in patients with CKD and in mice experiencing unilateral ureteral obstruction (UUO), which were strongly associated with the severity of renal fibrosis and renal functional impairment. UUO-induced renal inflammation and fibrosis were ameliorated by genetic deletion of NK-1R but aggravated by SP administration. In addition, pharmacological inhibition of NK-1R also attenuated renal inflammation and fibrosis. Mechanistically, our study showed that NK-1R was a direct target of the transcription factor activating enhancer-binding protein 4 (TFAP4). TFAP4 directly bound to the NK-1R promoter to promote its transcription. Furthermore, we also elucidated that SP/NK-1R signaling promoted JNK and p38 phosphorylation in renal tubular cells, whereby it modulated cell fate of TECs, including G2/M arrest, apoptosis, and profibrogenic/proinflammatory responses to consequently accelerate renal inflammation and fibrosis. Therefore, this study demonstrates that activation of the SP/NK-1R axis in tubular cells contributes to renal inflammation and fibrosis. Targeting this pathway may be a promising strategy for renal fibrosis and CKD treatment.

## Materials and methods

### Reagents

Substance P (SP, GC15649; GLPBIO, CA, USA), SR140333 (GC11256; GLPBIO), SP600125 (GC15344; GLPBIO) and SB239063 (GC10054; GLPBIO) were used. PBS was used as vehicle control for SP, and DMSO as a control for SR140333, SP600125, and SB239063.

### Human samples and clinical information

Human renal biopsies from patients living with CKD (stage 3–5, n = 21), together with 6 surgically removed adjacent non-tumor kidney tissues of individuals with renal cancer as relatively normal control and human serum samples from 28 CKD patients (stage 3–5) and 20 healthy persons were collected from the Seventh Affiliated Hospital, Sun Yat-sen University, Shenzhen, China. Clinical information and laboratory examination data of indicated individuals were collected. The clinical characteristics of patients with CKD are shown in **Supplementary Table S1**. The baseline features between patients with CKD and control individuals are shown in **Supplementary Table S2**. Human samples protocols gained approval from the Institutional Research Ethics Committee at the Seventh Affiliated Hospital, Sun Yat-sen University (Approval No. KY AF/SC-07/01.0) and informed consent was obtained from all individuals.

### Animals

We obtained 8–12-week-old wild-type C57BL/6J male mice from GemPharmatech Co. Ltd, Nanjing, China. NK-1R knockout (NK-1R^-/-^) C57BL/6J mice were also generated by GemPharmatech using the CRISPR-Cas9 system. The left ureter was ligated to construct the UUO mouse model according to a previous description (19). Mice were euthanized 7 or 14 days after UUO surgery. Animal experimental protocols gained approval from the Institutional Research Ethics Committee at the Sun Yat-sen University (Approval No. SYSU-IACUC-2022-000226) and were carried out following the relevant guidelines and the Guide for the Care and Use of Laboratory Animals (NIH publications Nos. 80-23, revised 1996).

### Histology and immunohistochemistry assay

After harvesting kidney tissues, Masson’s trichrome and periodic acid-Schiff (PAS) staining were performed to assess tubular/tubulointerstitial injury and fibrosis, respectively. Immunohistochemistry analyses were carried out using antibodies against NK-1R (NB300-119, diluted 1:400; Novus Biologicals, Littleton, CO, USA), SP (DF7522, 1:200; Affinity Biosciences, Jiangsu, China), Collagen1 (14695-1-AP, 1:400; Proteintech, Wuhan, China); F4/80 (70076, 1:400; Cell Signaling Technology, Beverly, MA, USA), α-SMA (19245, 1:200; Cell Signaling Technology); MCP-1(A7277, 1:2500; ABclonal Technology, Wuhan, China), TNF-α (A11534, 1:400; ABclonal Technology), TFAP4 (12017-1-AP, 1:100; Proteintech). The fibrotic-, NK-1R-, SP-, Collagen I-, F4/80-, α-SMA-, MCP-1-, TNF-α- and TFAP4-positive areas were measured in 10 randomly chosen high-power fields per kidney section using ImageJ software, version 1.53e (National Institutes of Health, Bethesda, USA).

### ELISA assay

Serum samples from both human and mice were collected for SP detection. SP level was determined with ELISA kits (Cusabio, Wuhan, China) in line with specific protocols.

### TUNEL assay

The ApopTag Plus Peroxidase *In Situ* Apoptosis Detection Kit (Chemicon, Temecula, CA, USA) was adopted for TUNEL assay in detecting apoptotic cell rate in line with specific protocols. Later, fluorescence microscopy was performed to determine the apoptotic cell number in 10 randomly chosen high-power fields per kidney section.

### RNA isolation and real-time quantitative PCR

This work utilized Trizol reagent (ThermoFisher Scientific, Darmstadt, Germany) to extract total RNA and reverse-transcribe it using the Quantitec Reverse Transcription Kit (Ruizhen Bio, Guangzhou, China) in line with the specific protocols. The mRNA expression levels were quantified by RT-qPCR using a SYBR Green PCR kit (Ruizhen Bio). All reactions were conducted in duplicate. The 2^-ΔΔCt^ approach was implemented to determine gene levels, with β-actin as a reference. Primers for β-actin, NK-1R, SP, Collagen1, a-SMA, MCP-1, TNF-α, CTGF, MMP9, and TFAP4 are described in **Supplementary Table S3**.

### Western blotting assay

RIPA buffer (Beyotime, Shanghai, China) supplemented with protease and phosphorylase inhibitors was added to lyse both kidney tissues and HK-2 cells. Later, proteins were separated through SDS-PAGE, followed by transfer on PVDF membranes. Thereafter, 5% BSA was added to block membranes under room temperature (RT) for a 1-h period, followed by primary antibody incubation, including NK-1R (ab183713, 1:1000; Abcam, Cambridge, MA, USA), SP (DF7522, 1:1000; Affinity Biosciences); α-SMA (19245, 1:5000), phosphorylated-p38 (4511, 1:1000), p38 (8690, 1:1000), phosphorylated-ERK (4370, 1:1000), ERK (4695, 1:1000), Cell Signaling Technology; phosphorylated-JNK (ab124956, 1:1000; Abcam), JNK (ab179461, 1:1000; Abcam); TFAP4 (12017-1-AP, 1:500; Proteintech), Collagen1 (14695-1-AP, 1:1000; Proteintech), and GAPDH (60004-1-Ig, 1:20000; Proteintech) under 4°C overnight. Afterward, HRP-labeled secondary antibodies (15015, 15014, diluted 1:10000; Proteintech) were added to further incubate membranes for 1 hour at RT. Protein detection was performed through WB Chemiluminescence Detection, with GAPDH being the endogenous reference.

### Cell culture and transfection

We cultivated HK-2 cells within DMEM/f12 containing 10% fetal bovine serum (FBS), 1% penicillin/streptomycin (P/S) under 5% CO2 and 37°C conditions. By adopting ClonExpress^®^ One Step Cloning Kit (Vazyme Biotech, Nanjing, China), overexpression plasmids pCDH-NK-1R and pCDH-TFAP4 were produced by inserting full-length NK-1R or TFAP4 in pCDH-CMV-MCS-EF1-Puro’s EcoR I/BamH I sites. For producing lentiviruses, the overexpression plasmid, psPAX2 was co-transfected with pMD2.G plasmid into HEK293T cells using Lipofectamine 2000 (Invitrogen, Carlsbad, CA, USA). Then, this work collected the lentiviral supernatant and preserved it under −80°C prior to analysis. Later, lentiviral supernatant that contained polybrene (8 μg/mL) was added to infect HK-2 cells, and then screened by puromycin (2 μg/mL) for obtaining HK-2 cells stably overexpressing NK-1R or TFAP4.

### Cell counting assay

We cultivated NK-1R-overexpressed HK-2 cells (5 × 10^4^) into the 12-well plate and then subjected to treatment with 20 μM SP with or without 10 μM SR140333 for 48 hours prior to calculating cell number with Countstar (ALIT Life Sciences, Shanghai, China).

### Colony formation assay

We maintained NK-1R-overexpressed HK-2 cells (5 × 10^2^) into the six-well plate for 1 day, and then subjected to treatment with 20 μM SP with or without 10 μM SR140333 for another 8 days. Methanol was added to fix colonies, followed by 0.1% crystal violet staining as well as counting.

### Flow cytometry

Apoptotic cell proportion and cell cycle distribution were evaluated by FCM. NK-1R-overexpressed HK-2 cells were incubated with the indicated treatment (20 μM SP, 10 μM SR140333, 5 μM SP600125, 2.5 μM SB239063) for 48 hours, and then evaluated by staining with Annexin V/DAPI staining using Annexin V-APC/DAPI Apoptosis Kit (Elabscience Biotechnology, Wuhan, China) or with propidium iodide (PI) by adopting Cell Cycle Analysis Kit (4A Biotech, Beijing, China). Thereafter, fluorescence-activated cell sorting (FACS) was carried out (CytoFLEX, Beckman Coulter, Miami, FL, USA).

### Luciferase report assay

Luciferase activities were determined by dual-luciferase reporter assay (Promega). *Renilla* luciferase that expresses pRL-TK (Promega) served as the endogenous reference for amending heterogeneities of transfection efficiency. To explore how TFAP4 affected the activity of the NK-1R promoter, 25 ng pRL-TK and 50 ng *firefly* luciferase reporter plasmid [p-(−1.5/+0.1k), p-mutA or p-mutB] and 150 ng pCDH-CMV-MCS-EF1-Puro or pCDH-TFAP4 were co-transfected into cells for 48 hours.

### ChIP assay

To confirm the interaction between proteins and target gene promoters, a ChIP assay was conducted as previously described (20) with some modifications. A 1% formaldehyde solution was added to cross-link HK-2 cells in a 10-cm dish for a 10-min period at RT and then collected by DTT solution. Subsequently, the cells were resuspended within SDS lysis buffer, followed by sonication 4°C for shearing DNA to 200–750 bp. After mixing with dilution buffer, the chromatin complexes were immunoprecipitated with 4 µg of anti-TFAP4 antibody (Proteintech) or isotype-matched rabbit control IgG (A7016; Beyotime) at 4°C overnight, followed by collection with 20 µL of Protein A/G MagBeads (Bimake) at 4°C for 2 hours. The bead-bound immunocomplexes were washed by pre-chilled IP lysis buffer, followed by elution with elution buffer and reverse crosslink of the DNA-protein. After purification of the immunoprecipitated DNA, semi-quantitative PCR and RT-qPCR were conducted by specific primers listed in **Supplementary Table S3**.

### Data extraction and processing

The gene expression profile (GSE66494) of human CKD kidneys was obtained in Gene Expression Omnibus (GEO, http://www.ncbi.nlm.nih.gov/geo/). Moreover, the R software limma package (version 4.1.1) was applied in identifying differentially expressed genes (DEGs) of CKD compared with non-CKD kidney tissues upon the thresholds of |log2 (Fold Change, FC)| >= 1 together with false discovery rate (FDR) *<* 0.05. Up-regulated DEGs were later enriched by a Kyoto Encyclopedia of Genes and Genomes (KEGG) analysis based on the Database for Annotation, Visualization, and Integrated Discovery (DAVID, https://david.ncifcrf.gov/). NK-1R co-expressed genes in GSE66494 were selected with cut-off of *p* < 0.05 and r >= 0.3 using Spearman′s correlation coefficient analysis. NK-1R potential transcriptional factors were predicted by ConTra v3 (http://bioit2.irc.ugent.be/contra/v3/) with a threshold of *p* < 0.05. Then, up-regulated DEGs, NK-1R co-expressed genes, and predicted NK-1R transcriptional factors were intersected.

### RNA-seq analysis

We added 20 μM SP or PBS to NK-1R-overexpressed HK-2 cells for a 48-h incubation period, followed by harvesting for RNA isolation using Trizol reagent. RNA preparation, library construction, and sequencing on the platform DNBSEQ (sequencing length, PE150) were conducted by the Beijing Genomics Institute (BGI, Shenzhen, China). Clean reads were obtained by eliminating raw sequencing reads, which were then mapped to the reference genome of Homo sapiens (GCF_000001405.39_GRCh38.p13) using HISAT (21). The expression levels of genes were quantified by RSEM (22) and DEGs between groups were selected according to DESeq2 (23) under the |log2 (FC)| >= 1 and FDR < 0.05. KEGG enrichment analysis of DEGs was based on the DAVID database.

### Statistics analysis

GraphPad Prism version 9.0.2 (GraphPad Software Inc., San Diego, CA, USA) was utilized for statistical analysis. Results were represented by mean ± SEM from three or more individual assays. Differences between the two groups were compared by unpaired Student′s t-test. Multiple comparisons among groups were made using one‐way ANOVA test followed by Bonferroni adjustment (assuming equal SDs) or Brown-Forsythe and Welch ANOVA tests followed by Dunnett T3 adjustment (assuming unequal SDs). For correlation analysis, a linear regression analysis was performed with Spearman′s correlation coefficients. *p* < 0.05 stood for statistical significance.

## Results

### Renal SP/NK-1R and serum SP levels are elevated in patients with CKD and correlated with severe renal fibrosis and declined kidney function

To assess the expression and cellular localization of SP and NK-1R, kidney biopsies were collected from 21 patients with CKD (stage 3-5) and 6 adjacent relatively normal kidney tissues from surgically removed renal cell carcinoma (RCC) tissues were obtained as controls.

**Figure 1:**
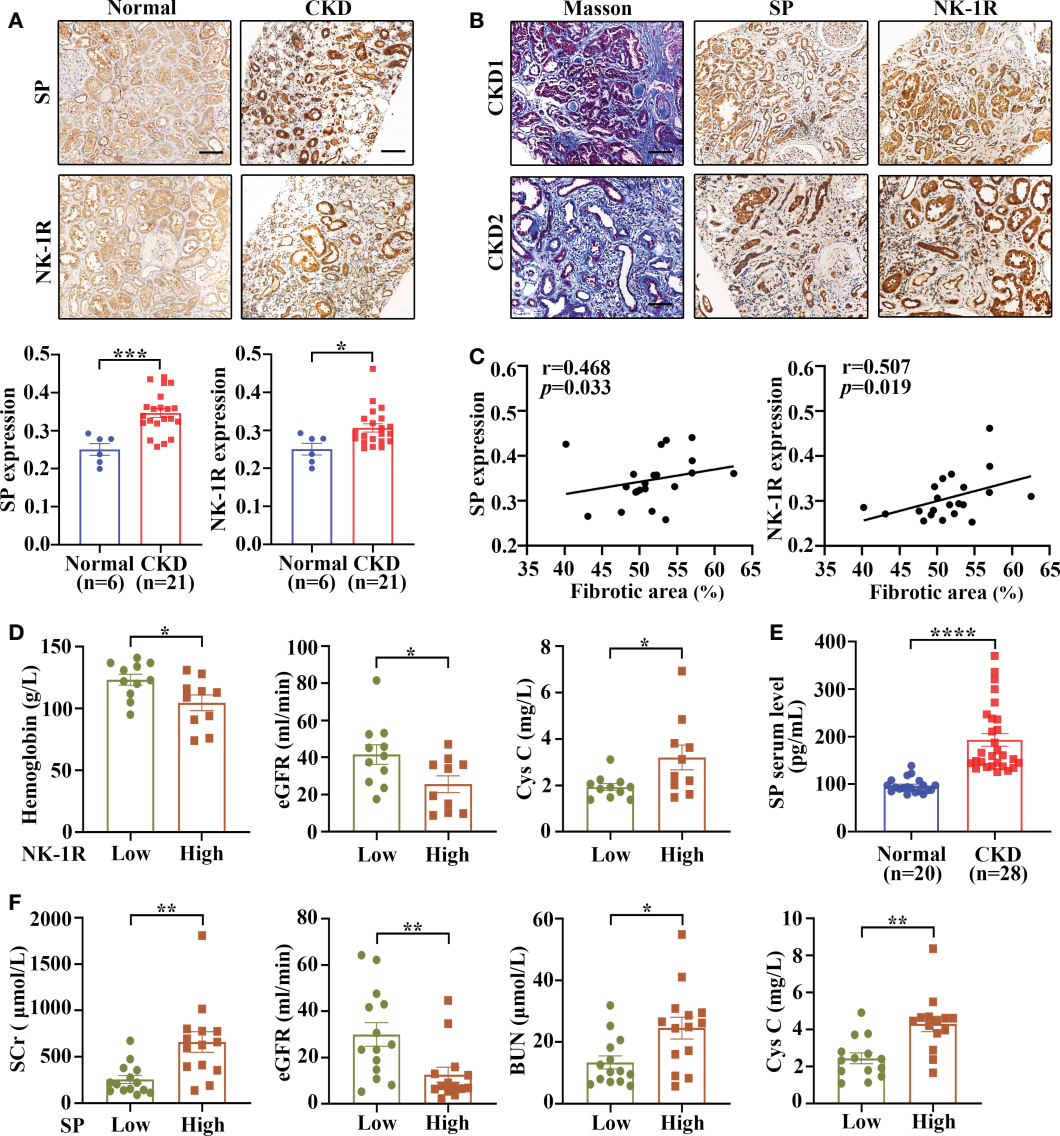
Renal SP/NK-1R and serum SP were up-regulated in patients with CKD and related to renal fibrosis and function. **(A)** The expression levels of NK-1R and SP were significantly increased in the fibrotic kidneys of patients with CKD, as assessed by immunohistochemistry staining. Representative sections (upper panels) and quantitative data (lower panels) are shown. **(B, C)** Spearman’s correlation coefficient analysis showed that the expression levels of renal NK-1R and SP were positively correlated with renal fibrotic extents in 21 CKD individuals. Representative sections of immunohistochemistry staining and Masson’s trichrome are exhibited in **(B)**. **(D)** Correlation between clinical characteristics and high/low renal expression of NK-1R in 21 CKD patients. The median NK-1R level was selected as the cut-off value for separating high from low NK-1R expression groups. **(E)** ELISA analysis displayed increased serum SP levels in CKD patients. **(F)** Correlation between clinical characteristics and high/low serum SP levels in 28 CKD patients. Samples were classified into high and low SP level groups using the cutoff of median SP level. Scale bar, 100 µm. Data are shown as mean ± SEM. *p < 0.05; **p < 0.01; ***p < 0.001; ****p < 0.0001.

Immunohistochemistry demonstrated that both SP and NK-1R were predominantly localized to renal TECs, and their expression notably increased in CKD kidneys compared with control kidneys (**Figure 1A**). In addition, Masson’s trichrome staining showed that CKD kidney tissues with higher SP and NK-1R expression exhibited more severe tubulointerstitial fibrosis (**Figure 1B**). Spearman′s correlation analysis revealed that fibrotic extents in CKD kidneys were positively correlated with renal NK-1R (r = 0.507, *p* = 0.019) or SP (r = 0.468, *p* = 0.033) expression levels (**Figure 1C**). Based on the median NK-1R expression level in CKD kidneys, the samples were classified into low- and high-NK-1R groups. Relationships between renal NK-1R expression and the clinical characteristics of patients with CKD were shown in **Supplementary Table S4**. Compared to CKD patients with low NK-1R expression, CKD patients with high NK-1R expression had lower hemoglobin (Hb, *p =* 0.023) levels, decreased estimated glomerular filtration rates (eGFR, *p=*0.035), and increased serum cystatin c (Cys C, *p =* 0.043) levels (**Figure 1D**). Meanwhile, enzyme-linked immunosorbent assay (ELISA) revealed that serum SP levels were up-regulated in patients with CKD (**Figure 1E**) and were correlated to clinical features (**Supplementary Table S5**). Compared with CKD patients with low serum SP levels, those with high serum SP levels displayed worse renal function as indicated by elevated levels of serum creatinine (Scr, *p =* 0.003), blood urea nitrogen (BUN, *p* = 0.012), Cys C (*p =* 0.0011) and decreased eGFR (*p =* 0.008) (**Figure 1F**). Thus, our results suggest that renal SP and NK-1R expression are augmented and strongly correlated with renal fibrotic extent and renal functional impairment in patients with CKD.

### Renal SP and NK-1R expression are upregulated in the mouse UUO kidneys

**Figure 2:**
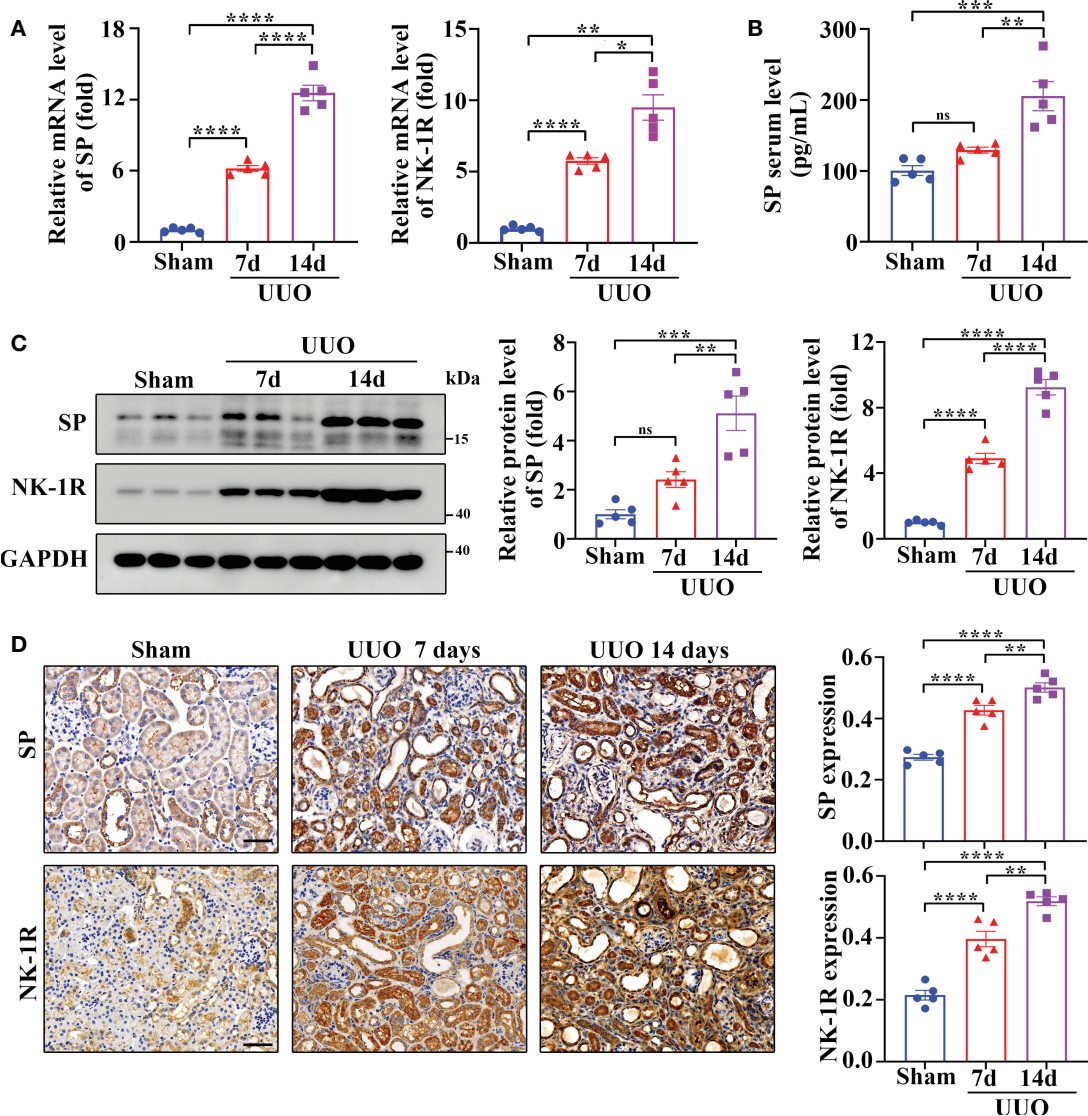
Renal SP/NK-1R and serum SP were overexpressed in a mouse unilateral ureteral obstruction (UUO) model. **(A)** RT-qPCR exhibited increased levels of SP and NK-1R mRNA in cortical lysates of kidneys on days 7 and 14 after UUO. **(B)** ELISA analysis displayed elevated serum SP levels on days 7 and 14 after UUO. **(C, D)** Western blot analysis and immunochemistry staining showed that the protein levels of renal SP and NK-1R in cortical tissues were up-regulated on days 7 and 14 after UUO. Representative images (left panels) and quantitative data (right panels) are shown. Scale bar, 50 µm. Data are shown as mean ± SEM from groups of five mice. *p < 0.05; **p < 0.01; ***p < 0.001; ****p < 0.0001; ns, not significant.

UUO is a widely used as an animal model of renal fibrosis, which exhibits renal inflammation and fibrosis similar to CKD (24). As revealed by RT-qPCR assay, SP and NK-1R mRNA levels were significantly elevated in kidneys 7 and 14 days after UUO surgery (**Figure 2A**), and the serum levels of SP protein increased time-dependently during the UUO injury (**Figure 2B**). In addition, both Western blotting and immunohistochemistry detected that compared to sham-operated kidneys, the UUO kidneys showed a marked increase in the expression of SP and NK-1R in a time-dependent manner (**Figures 2C, D**). Consistent with the observations in human CKD, SP and NK-1R were mainly expressed in TECs of UUO kidneys (**Figure 2D**). These findings indicate that tubular SP and NK-1R may be involved in regulation of renal fibrosis.

### Genetic deletion of NK-1R protects UUO mice from the development of renal inflammation and fibrosis

**Figure 3:**
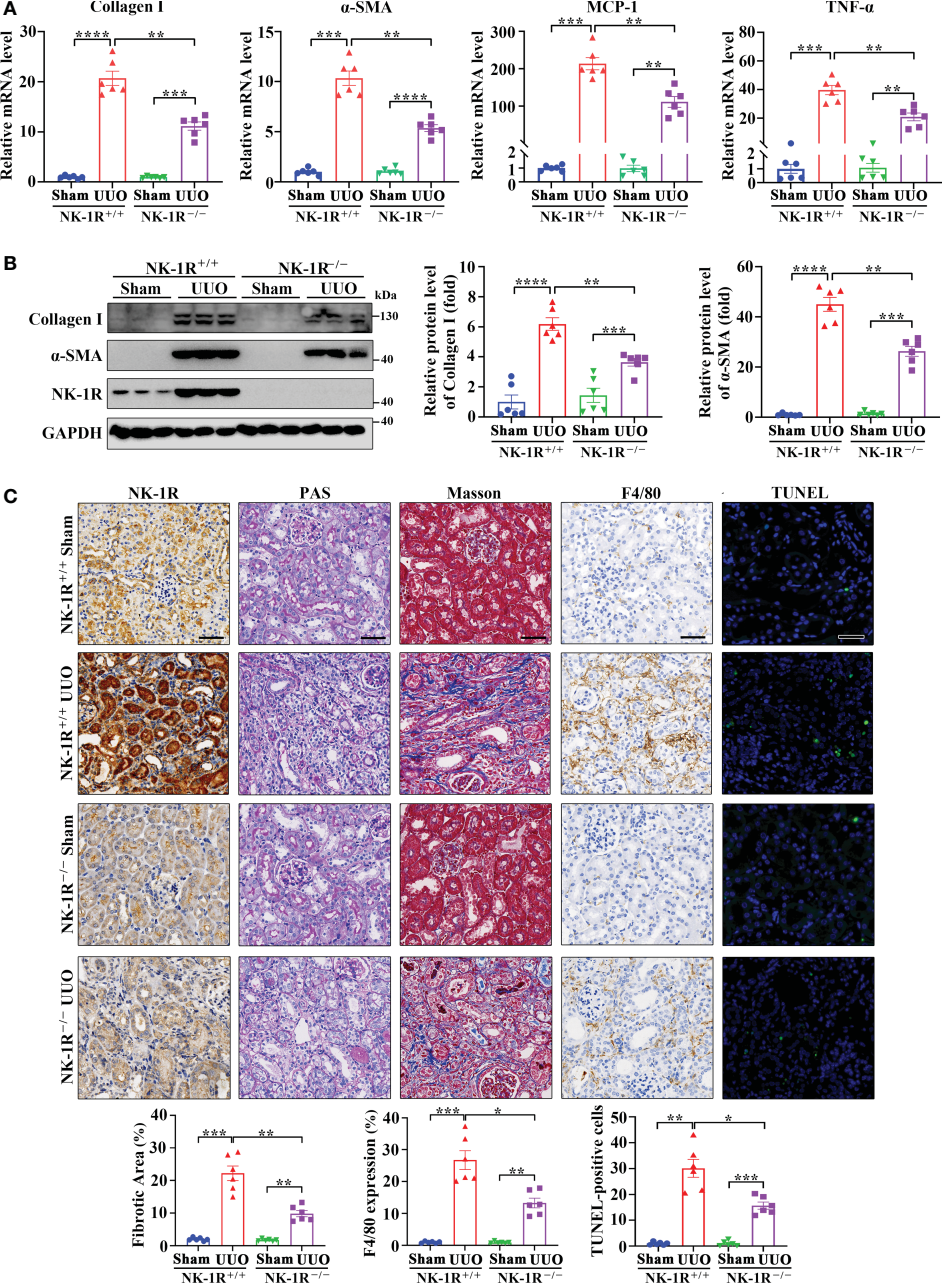
Genetic deletion of NK-1R alleviated UUO-induced renal fibrosis, inflammation, and apoptosis. **(A, B)** RT-qPCR **(A)** and Western blotting **(B)** displayed the reduced mRNA **(A)** and protein **(B)** levels of Collagen I, α-SMA, MCP-1, and TNF-α in renal cortical tissues of NK-1R knockout (NK-1R^-/-^) UUO mice, compared with NK-1R wildtype (NK-1R^+/+^) UUO mice. **(C)** Immunochemistry staining of NK-1R and F4/80, PAS, Masson’s trichrome, and TUNEL analysis showed that the deletion of NK-1R attenuated UUO-induced renal fibrosis, infiltration of F4/80-positive inflammatory cells, and apoptosis. For **(A–C)**, mouse kidneys were excised on day 14 after UUO. Analysis of TUNEL-positive cells was counted by fluorescence microscopy in ten randomly selected high-power fields per kidney section. Scale bar, 50 µm. Data are shown as mean ± SEM from groups of six mice. *p < 0.05; **p < 0.01; ***p < 0.001; ****p < 0.0001.

To identify the role of the SP/NK-1R axis in renal fibrosis, NK-1R knockout (NK-1R^-/-^) mice were generated (**Supplementary Figure 1A**) and subjected to UUO for 14 days. Compared with NK-1R^+/+^ mice, NK-1R^-/-^ counterparts showed alleviated renal inflammation as showed by decreases in mRNA and protein levels of proinflammatory factors monocyte chemoattractant protein-1 (MCP-1) and TNF-α (**Figure 3A** and **Supplementary Figure 1B**). Consistently, immune cell infiltration and F4/80 positive macrophage numbers were reduced in NK-1R^-/-^ kidneys compared with NK-1R^+/+^ kidneys as shown by PAS and F4/80 staining respectively (**Figure 3C**).

As inflammation is closely associated with renal fibrosis (25), we therefore examined renal fibrosis in NK-1R^-/-^ mice. The mRNA and protein levels of α-smooth muscle actin (α-SMA) and collagen I were lower in NK-1R^-/-^ kidneys than in NK-1R^+/+^ kidneys following UUO (**Figures 3A, B** and **Supplementary Figure 1B**). Masson’s trichrome showed decreased collagen matrix deposition in NK-1R^-/-^ UUO kidneys (**Figure 3C**). These results suggest that deletion of NK-1R attenuates UUO-induced fibrosis.

PAS showed that deletion of NK-1R effectively lessened UUO-induced tubule injury (**Figure 3C**). Renal cell apoptosis has been implicated in the progression of renal fibrosis. Interestingly, TUNEL positive cell numbers were increased by UUO in NK-1R^+/+^ mice, which were decreased in NK-1R^-/-^ mice (**Figure 3C**). These results suggest that deletion of NK-1R attenuates UUO-induced renal inflammation, apoptosis, and fibrosis.

### Administration of SP promotes renal inflammation and fibrosis induced by UUO

**Figure 4:**
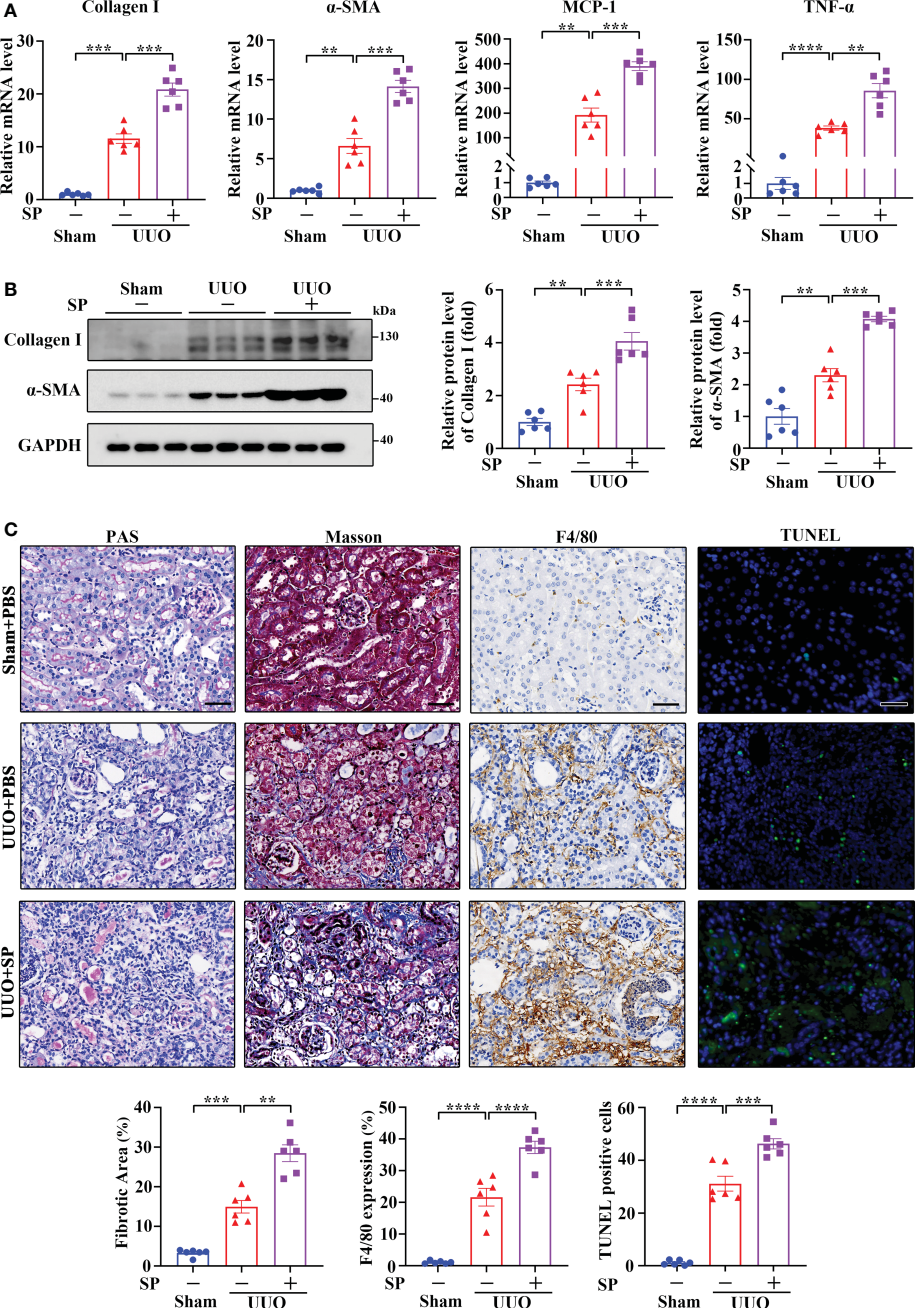
Administration of SP exacerbated UUO-induced renal fibrosis, inflammation, and apoptosis. **(A–C)** RT-qPCR **(A)**, Western blotting **(B)**, PAS, Masson’s trichrome, F4/80 immunochemistry staining, and TUNEL analysis **(C)**. Results showed that SP administration in established UUO mice enhanced the expression of Collagen I, α-SMA, MCP-1, and TNF-α at mRNA and/or protein levels, which was accompanied by aggravated renal fibrosis, infiltration of F4/80-positive inflammatory cells, and apoptosis. For **(A–C)**, sham and UUO mice were subjected to vehicle (PBS) or SP (40 nM/kg body weight) by tail vein injection three times a week for 14 days. The quantitative analysis of TUNEL-positive cells was achieved by fluorescence microscopy in 10 randomly chosen high-power fields per kidney section. Scale bar, 50 µm. Data are shown as mean ± SEM from groups of six mice. **p < 0.01; ***p < 0.001; ****p < 0.0001.

It is well accepted that SP signals primarily through NK-1R. To further explore the role of the SP/NK-1R axis in kidney disease, SP, an agonist of NK-1R, was administered to mice after UUO surgery. As shown by RT-qPCR, Western blotting and immunohistochemistry, administration of SP obviously augmented the mRNA and protein levels of proinflammatory/profibrogenic factors in kidneys 14 days after UUO (**Figures 4A, B** and **Supplementary Figure 2**). Moreover, SP also significantly increased the influx of inflammatory cells, F4/80-positive macrophages, TUNEL-positive apoptotic cells and renal fibrosis (**Figure 4C**). These results confirm that activation of the SP/NK-1R axis amplifies renal inflammation, apoptosis, and fibrosis induced by UUO.

### Treatment with a pharmacological NK-1R inhibitor attenuates renal inflammation and fibrosis induced by UUO

**Figure 5:**
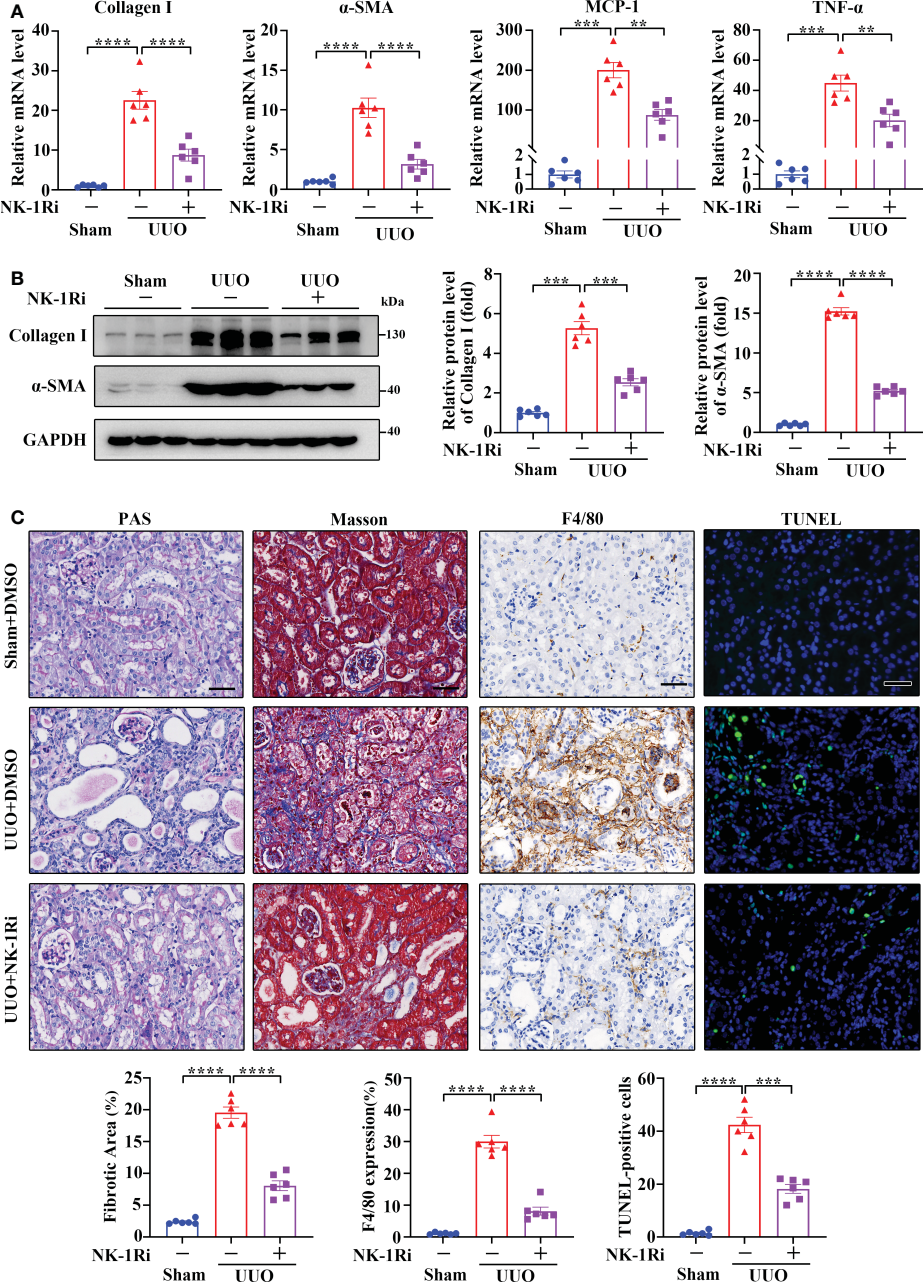
Inhibition of NK-1R with a pharmacological antagonist attenuated UUO-induced renal fibrosis, inflammation, and apoptosis. **(A, B)** RT-qPCR **(A)** and Western blotting **(B)** showed that the inhibition of NK-1R antagonized the UUO-induced increase in Collagen I, α-SMA, MCP-1, and TNF-α at the mRNA and/or protein levels in renal cortical tissues on day 14 after UUO. For B, representative images (left panels) and quantitative data (right panels) are shown. **(C)** PAS, Masson’s trichrome, F4/80 immunochemistry staining, and TUNEL analysis displayed that the inhibition of NK-1R impeded renal fibrosis, infiltration of F4/80-positive inflammatory cells, and apoptosis mediated by UUO surgery. Representative images (upper panels) and quantitative data (lower panels) are shown. For **(A–C)**, sham and UUO mice were administered intraperitoneally with vehicle (DMSO) or a specific NK-1R inhibitor (NK-1Ri) SR140333 (1 mg/kg body weight) every day for 14 days. + or −, with (+) or without (−) the indicated treatment. TUNEL-positive cells were counted by fluorescence microscopy in ten randomly selected high-power fields per kidney section. Scale bar, 50 µm. Data are shown as mean ± SEM from groups of six mice. **p < 0.01; ***p < 0.001; ****p < 0.0001.

To examine if targeting NK-1R has a therapeutic effect on renal fibrosis, we treated UUO mice with an NK-1R-specific pharmacologic inhibitor, SR140333 (NK-1Ri). Administration of NK-1Ri significantly down-regulated expression of profibrogenic and proinflammatory factors in kidneys 14 days after UUO surgery (**Figures 5A, B** and **Supplementary Figure 3**). In addition, both histological and TUNEL analyses also detected the amelioration of tubule injury, renal inflammation, and fibrosis, as well as the reduction of apoptotic cell number, in mice treated with NK-1Ri (**Figure 5C**). These results unravel the promising therapeutic effects of pharmacological inhibition of NK-1R on progressive renal fibrosis.

### TFAP4 is up-regulated in fibrotic kidneys and transcriptionally activates NK-1R expression

**Figure 6:**
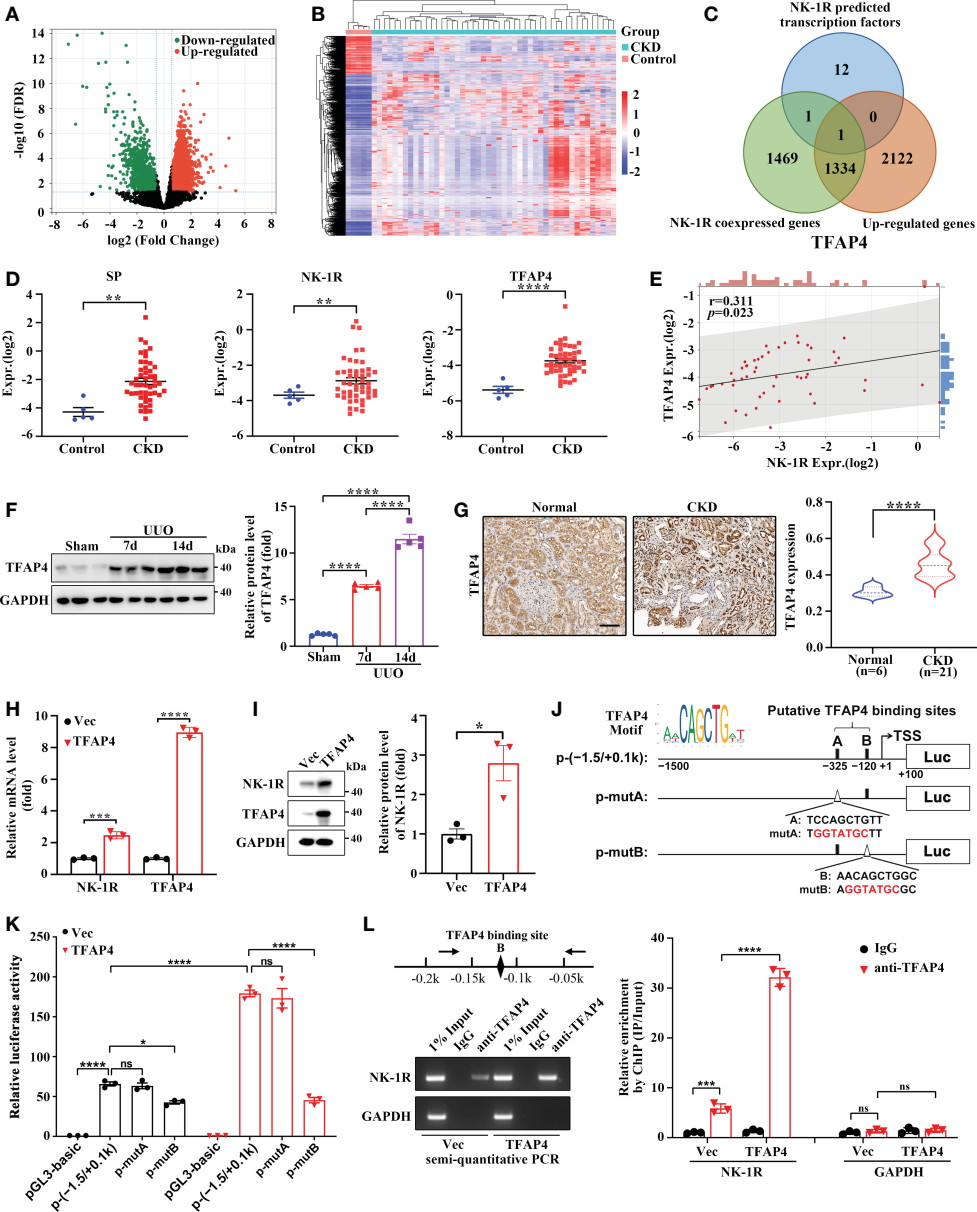
TFAP4 is dysregulated in fibrotic kidneys and transcriptionally activates NK-1R expression. **(A, B)** The volcano plot **(A)** and heatmap **(B)** of DEGs between CKD and non-CKD patients from GSE66494 with the threshold of FDR < 0.05 and |log2 (Fold Change)| > = 1. **(C)** Venn diagram of ConTra v3-predicted NK-1R transcription factors overlapping NK-1R co-expressed genes and up-regulated genes in GSE66494. **(D)** The mRNA levels of renal NK-1R, SP, and TFAP4 increased in CKD patients based on GSE66494. **(E)** Correlation between the expression of NK-1R and TFAP4 in GSE66494. **(F, G)** Western blotting **(F)** and immunochemistry staining **(G)** showed the up-regulated expression of renal TFAP4 protein in UUO mice and CKD patients, respectively. **(H, I)** Real-time quantitative PCR **(H)** and Western blotting **(I)** showed that the stable overexpression of TFAP4 increased NK-1R mRNA and protein levels in HK-2 cells. **(J, K)** Overexpression of TFAP4 enhanced the activity of the NK-1R promoter, whereas the mutation of the potential TFAP4 binding site B but not A reduced its activity. Schematic diagram for firefly luciferase reporter plasmids containing -1.5 to +0.1 k region of NK-1R is shown. Potential TFAP4 binding sites (BS) in the NK-1R promoter are depicted as a close rectangle A/B, and mutant TFAP4 BS are depicted as an open triangle A/B. **(L)** ChIP assay showed that TFAP4 directly interacted with the NK-1R promoter *in vivo*. The antibody-precipitated DNA was amplified by semi-quantitative PCR for 30 cycles (left) and real-time quantitative PCR (right). The promoter of GAPDH was used as a negative control. Data are presented as mean ± SEM. *p < 0.05; **p < 0.01; ***p < 0.001; ****p < 0.0001; ns, not significant.

To determine the regulatory mechanism of UUO-induced NK-1R expression, we analyzed the expression array data (GSE66494) of kidney biopsies from patients with CKD (**Figures 6A, B**). In order to identify the potential transcriptional factors that enhance NK-1R expression, we first selected 3457 up-regulated genes (log2 FC >= 1 and FDR < 0.05). Next, we calculated the Spearman′s correlation between NK-1R and other genes expression in GSE66494 and generated 2805 NK-1R coexpressed genes (r >= 0.3, *p* < 0.05). Last, 14 potential transcription factors were predicted by ConTra v3 to bind to the NK-1R promoter. TFAP4 was the only one that was found in all three sets of genes and thus was selected for further investigation (**Figures 6C–E**). Interestingly, TFAP4 mRNA levels increased in CKD along with elevated SP and NK-1R mRNA levels (**Figure 6D**).

TFAP4 protein expression was also increased in mouse UUO kidney (**Figure 6F**) and in patients with CKD (**Figure 6G**). TFAP4 expression in the CKD kidney was positively correlated to fibrotic extent (r = 0.46, *p* = 0.04) as well as NK-1R expression (r = 0.52, *p* = 0.02) (**Supplementary Figures 4A, B**). We then explored whether TFAP4 regulates NK-1R expression in HK-2 cells. As shown in **Figures 6H, I**, TFAP4 overexpression stimulated NK-1R expression at both mRNA and protein levels. As predicted by JASPAR (26), the 1500-bp sequence upstream of TSS of the NK-1R gene contains two potential TFAP4 binding sites (site A and B) **(Figure 6J)**. This sequence displayed visible promoter activity, which was enhanced by TFAP4 overexpression and suppressed by mutation in the site B but not the site A (**Figure 6K**). Consistently, ChIP assays demonstrated the binding of TFAP4 to the NK-1R promoter (**Figure 6L**). Thus, these findings suggest that TFAP4 regulates NK-1R transcription by binding to the NK-1R promoter directly.

### NK-1R promotes renal inflammation and fibrosis *via* the JNK and p38 pathway

**Figure 7:**
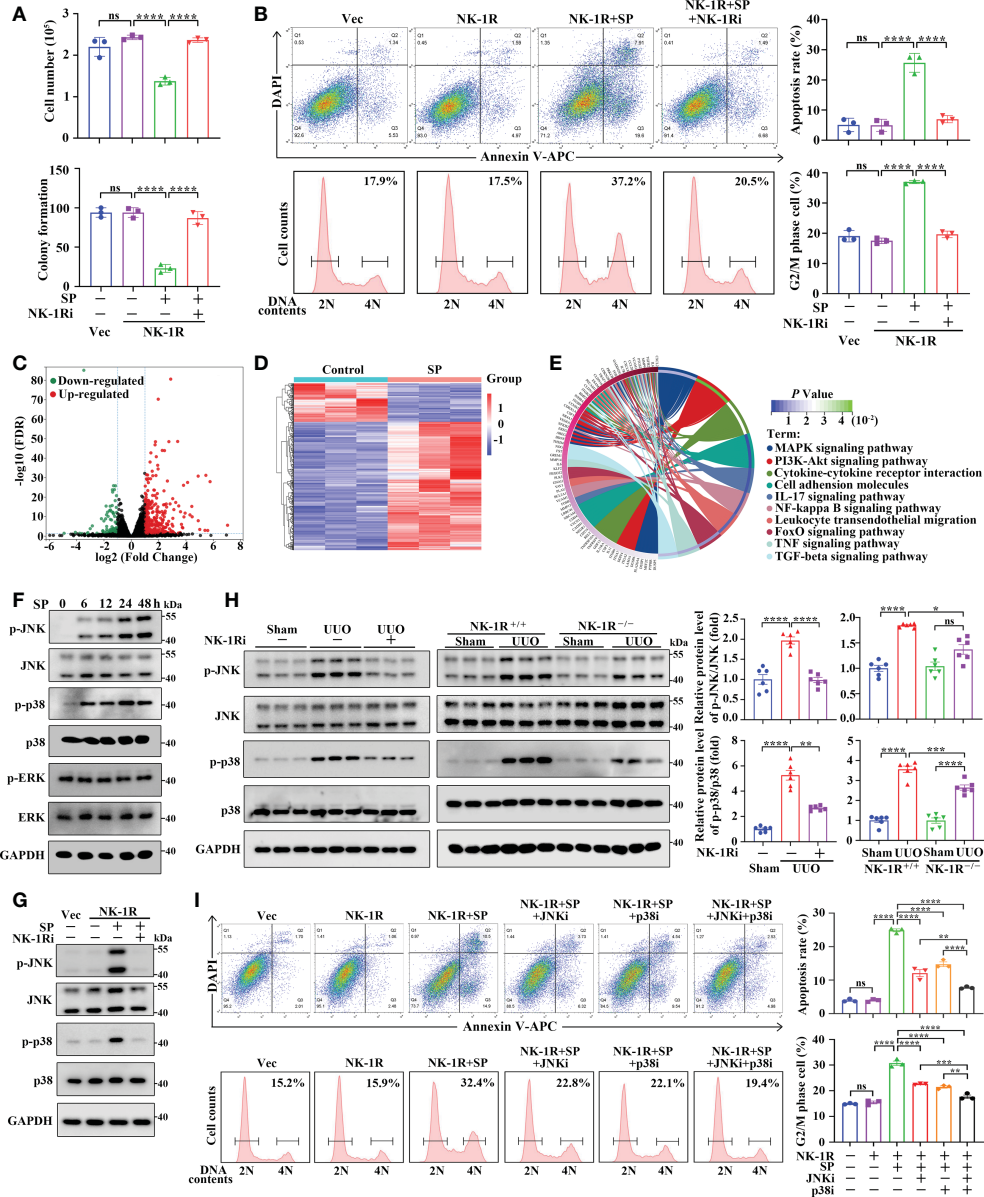
SP mediates renal inflammation and fibrosis through an NK-1R-dependent JNK/p38 mechanism *in vivo* and *in vitro*. **(A, B)** The NK-1R inhibitor abrogated the suppressive effect of SP on cell growth and colony formation **(A)** and abolished SP-induced apoptosis and G2/M arrest **(B)** in NK-1R-overexpressed HK-2 cells. For B, representative images (left panels) and quantitative data (right panels) are shown. **(C, D)** The volcano plot **(C)** and heatmap **(D)** of differentially expressed genes (DEGs) between NK-1R-overexpressed HK-2 cells treated with SP or its control using the criteria of FDR < 0.05 and |log2 (Fold Change)| > = 1. **(E)** The circos plot for the KEGG enrichment of indicated DEGs. **(F, G)** Western blotting showed a time-dependent increase in phosphorylated JNK and p38 but not ERK levels in NK-1R-overexpressed HK-2 cells upon SP incubation and that treatment with the NK-1R inhibitor blocked SP-induced increases in the expression of phosphorylated p38 and JNK. **(H)** Inhibition of NK-1R using both an NK-1R inhibitor and genetic knock-out strategies impaired the UUO-mediated elevation of phosphorylated JNK and p38 levels in mouse kidneys. **(I)** p38- or JNK-specific inhibitors or their combination significantly attenuated SP-stimulated G2/M arrest and apoptosis. For H and I, representative images (left panels) and quantitative data (right panels) are shown. Data are presented as mean ± SEM. *p < 0.05; **p < 0.01; ***p < 0.001; ****p < 0.0001; ns, not significant.

We then examined the machanisms through which the SP/NK-1R axis regulates renal inflammation and fibrosis in HK-2 cells stably overexpressing NK-1R (**Supplementary Figure 5A**). We found that addition of SP significantly suppressed the growth and colony formation of NK-1R-overexpressing HK-2 cells, whereas, these suppressive effects were reversed by the NK-1R-specific pharmacological inhibitor (**Figure 7A** and **Supplementary Figure 5B**). In addition, SP administration induced apoptosis and G2/M arrest in NK-1R-overexpressing HK-2 cells, which was also blocked by inhibition of NK-1R (**Figure 7B**). Interestingly, in SP-treated HK-2 cells that stably overexpressed NK-1R, the expression levels of profibrogenic factors, including connective tissue growth factor (CTGF) and MMP9, were obviously elevated, but these were markedly antagonized by NK-1R inhibition (**Supplementary Figure 5C**). Collectively, SP/NK-1R axis activation promoted apoptosis, G2/M arrest and profibrogenic factor expression in TECs.

To explore the regulatory mechanisms of SP/NK-1R axis in HK-2 cell activities, we treated the stable NK-1R-overexpressing HK-2 cells with and without SP. RNA-seq identified that 399 DEGs were detected in SP-treated cells compared with PBS-treated (control) cells with 307 being up-regulated and 92 down-regulated (|log2 FC| >= 1 and FDR < 0.05) (**Figures 7C, D**). KEGG analysis revealed the major enrichment of DEGs in the MAPK pathway, cytokine–cytokine receptor interaction, and PI3K-Akt pathway (**Figure 7E**). It has been well documented that MAPK signaling contributes to inflammation, apoptosis, and fibrosis. We then investigated whether the MAPK pathway was altered by SP/NK-1R activation in HK-2 cells. Phosphorylation of p38 and JNK, but not ERK, was highly up-regulated time-dependently by SP treatment in NK-1R-overexpressing HK-2 cells (**Figure 7F**). Furthermore, pharmacologically inhibiting NK-1R drastically repressed SP-induced JNK and p38 phosphorylation (**Figure 7G**). Consistent with the *in vitro* results, the increased p38 and JNK phosphorylation in UUO kidneys was attenuated by pharmacological inhibition or genetic deletion of NK-1R (**Figure 7H**).

To confirm the role of JNK and p38 in SP/NK-1R-mediated renal fibrosis, two inhibitors, namely, SP600125 (the JNK inhibitor) and SB203580 (the p38 inhibitor) were used to further assess whether MAPKs mediate the effects of the SP/NK-1R signaling on G2/M arrest, apoptosis and expression of profibrogenic genes. Results showed that treatment with SP600125 or SB203580 partially antagonized the stimulatory effects of SP on apoptosis and G2M arrest as well as profibrogenic gene levels in NK-1R-overexpressing HK-2 cells. Of note, the combined use of p38 and JNK inhibitors further inhibited SP-induced G2M arrest, apoptosis, and profibrogenic gene production (**Figure 7I** and **Supplementary Figure 5D**). Collectively, these results suggest that the SP/NK-1R axis may promote apoptosis and G2/M arrest as well as profibrogenic gene levels by activating the JNK and p38/MAPK pathways in TECs.

## Discussion

**Figure 8:**
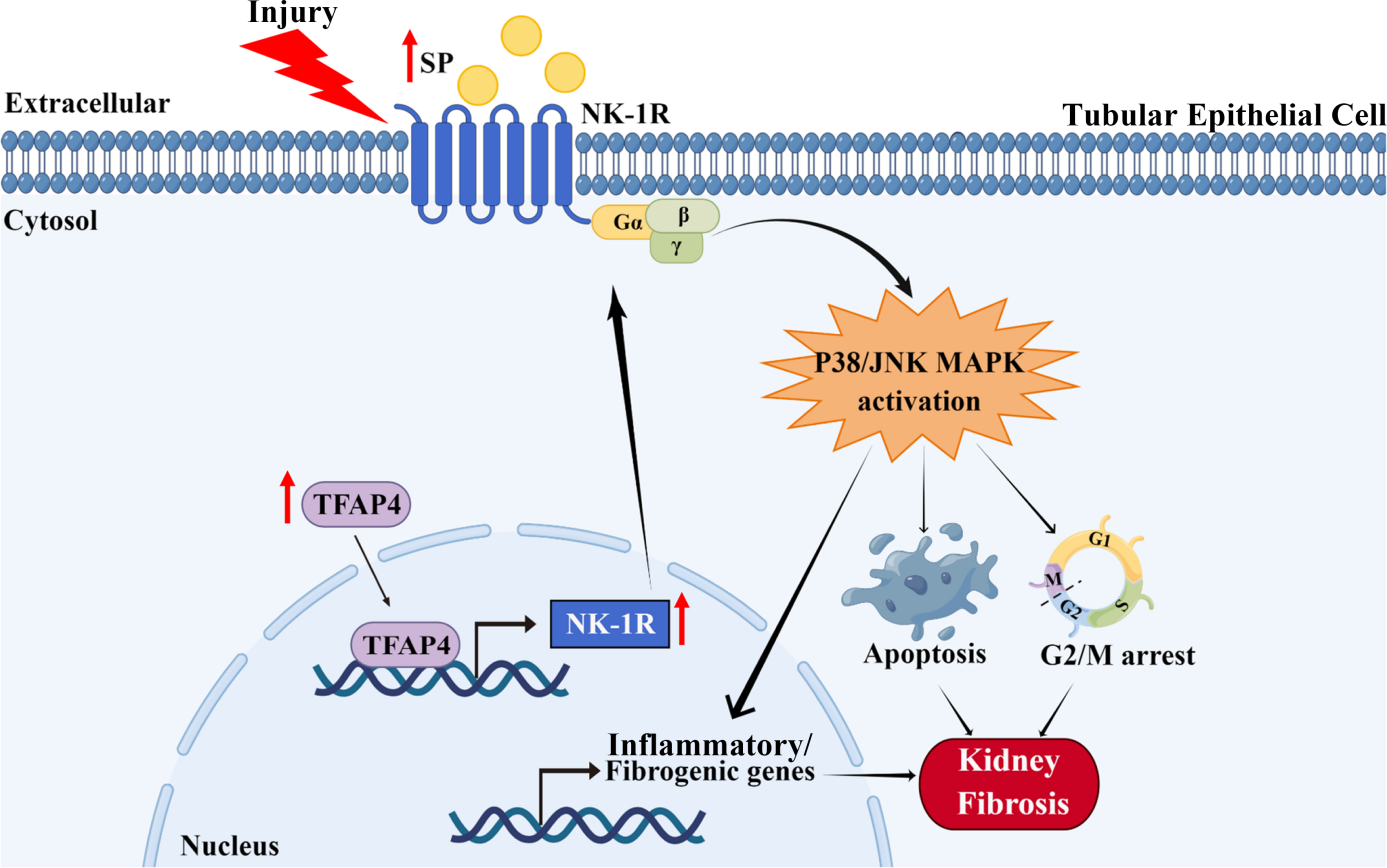
Schematic diagram generated by Figdraw for the proposed mechanism by which the SP/NK-1R axis contributes to the pathogenesis of renal fibrosis *via* modulating inflammatory responses and cell fate of tubular epithelial cells through JNK/p38 signaling pathway in CKD.

As a prevalent pathological hallmark of end-stage kidney disorders irrespective of the initial cause, renal fibrosis is featured with excessive ECM accumulation upon renal injury, leading to the destruction of normal renal structure and eventual kidney failure (27). Hence, renal fibrosis is well associated with renal dysfunction and is a poor prognostic indicator (28). Increasing evidence has revealed that TECs play an indispensable role in both initial and progressive stages of kidney fibrosis by undergoing various functional changes (such as programmed cell death, senescence, and cell cycle arrest) after injury, resulting from deregulation of pivotal factors/signaling pathways (26). Therefore, further investigation of the molecular mechanism underlying renal TECs abnormity is vital for understanding the pathogenesis of renal fibrosis. The SP/NK-1R pathway is related to the pathogenesis of diverse diseases, such as neurological, cardiovascular, and gastrointestinal diseases (13), as well as myocardial and liver fibrosis (18, 29). Nevertheless, the impact of the SP/NK-1R pathway on renal TEC dysfunction and consequent renal fibrosis remains unexplored. In the present study, as outlined in **Figure 8**, we identified that SP/NK-1R axis activation, arising from elevated SP and NK-1R levels upon injury, could augment renal cell apoptosis, G2/M arrest, proinflammatory/profibrogenic factors production, resulting in progressive renal fibrosis. Mechanistically, we found that NK-1R was a direct target of TFAP4 and TFAP4 promoted NK-1R transcription by binding to the NK-1R promoter. Furthermore, we also uncovered that SP acted *via* the NK-1R to activate the JNK and p38 pathways to induce G2/M arrest, apoptosis, and expression of proinflammatory/profibrogenic genes. More importantly, we also provided the first evidence for targeting the SP/NK-1R axis as a novel therapy for the patients with CKD.

Previous studies have well elucidated the extensive involvement of the SP/NK-1R axis in physiological or pathological processes related to central and peripheral nervous systems (e.g., pain, emesis, and neurodegenerative diseases). However, increasing evidence has also shown the expression of SP and NK-1R in non-neuronal tissues and cells (e.g., the liver, lung, and immune, epithelial, and endothelial cells) (30, 31). Indeed, NK-1R is found to promote inflammation, apoptosis, and fibrogenesis by associating with its ligand SP (15). It is reported that SP can bind to NK-1R to activate cardiac mast cells to increase TNF-α and MMPs expression and promote myocardial remodeling upon aortocaval fistula-induced volume overload (16). It can also provoke HSC/cholangiocyte senescence and liver cell apoptosis by stimulating TGF-β1 secretion after liver injury, leading to liver fibrosis (16, 18). Nevertheless, whether SP/NK-1R regulates renal fibrosis remains unknown.

In this study, we identified that expression of SP and NK-1R was significantly increased in the fibrotic kidney in patients with CKD and in mice after UUO and was positively correlated with the fibrotic extent in the renal interstitium. Serum SP levels were also increased in CKD individuals and UUO mice. More importantly, CKD patients with high NK-1R expression or SP levels exhibited lower eGFR, indicating that the activation of SP/NK-1R may contribute to the progression of CKD. Further investigations revealed that both genetic deletion and pharmacological inhibition of NK-1R remarkably impeded UUO-induced proinflammatory/profibrogenic responses as indicated by decreased macrophage infiltration, TNF-α/MCP-1/α-SMA/Collagen I levels, and renal cell apoptosis, whereas SP administration significantly enhanced these inflammatory and fibrogenic responses. Altogether, we find that the SP/NK-1R pathway mediates renal fibrosis progression.

Although SP and NK-1R levels are generally up-regulated in response to different injuries in diverse cell types, the regulatory mechanism of SP/NK-1R expression remains unclear. It has been reported that TFAP4, a member of the basic helix–loop–helix leucine-zipper (bHLH-LZ) family (32), is associated with c-Myc-mediated renal fibrosisby by inducing integrin αv-mediated TGF-β signaling (33). However, no direct evidence has yet revealed the regulatory role of TFAP4 in renal fibrosis. Hyperactivation of TFAP4 has been observed in various human malignancies (e.g., intestinal, lymphoid and liver cancer) (34–36). In the present study, we identified that increased renal TFAP4 levels were closely correlated to the fibrotic index and NK-1R levels in patients with CKD. Moreover, KEGG enrichment analysis suggested that TFAP4 might be associated with multiple signaling pathways (**Supplementary Figure 4C**), such as neuroactive ligand-receptor interaction, MAPK, and Wnt signaling pathways. More importantly, we uncovered that NK-1R expression was regulated by TFAP4 as TFAP4 could bind to the promoter region of NK-1R to regulate its transcription. In the present study, therefore, TFAP4 may be a regulatory mechanism responsible for NK-1R overexpression in renal fibrosis.

Renal fibrosis involves different types of resident cells, including renal TECs and myofibroblasts. It is reported that apoptosis and cell cycle arrest of renal TECs initiate adaptive repair in response to kidney injury (37). Previous studies have demonstrated that maladaptive cell death results in the loss of resident renal TECs and that apoptotic tubule communicates with adjacent profibrogenic cell types (e.g., fibroblasts, myofibroblasts), eventually promoting fibrosis (37). Of these processes, the G2/M phase arrest of renal TECs after kidney injury contributes to persistent transcription of profibrogenic factors such as CTGF and Collagen I (38, 39). Given that SP and NK-1R were mainly expressed in renal TECs, we used HK-2 cells that stably overexpressed NK-1R to further investigate the relationship between SP/NK-1R and tubular injury as well as fibrogenesis *in vitro*. We found that SP treatment suppressed the growth and induced G2/M arrest, apoptosis, and profibrogenic factor production in NK-1R-overexpressing HK-2 cells. Correspondingly, the above effects of SP were almost abolished by a specific NK-1R antagonist, indicating that SP functioned through NK-1R activation. We thus propose that the SP/NK-1R axis may contribute to renal fibrosis at least partly by provoking phenotypic changes in tubular epithelium cells.

To explore the mechanism underlying SP/NK-1R-mediated tubular injury and fibrogenesis, RNA-seq and KEGG pathway enrichment analyses were performed. We found that the SP/NK-1R axis was associated with several signaling pathways closely related to renal fibrosis, such as MAPK and PI3K-Akt. The MAPK kinase superfamily includes four typical subgroups, including JNK, p38, ERK1/2, and ERK5, and it is worth noting that the first three members are dominant responders to extracellular and intracellular stresses (40). Extensive studies have suggested that MAPKs participate in multiple events such as cell differentiation, cell death, and inflammation, and play a critical role in various diseases (41). We found that SP treatment induced the phosphorylation of p38 and JNK but not ERK1/2 *in vitro*, which was completely blocked by the NK-1R antagonist. In addition, the stimulatory effect of UUO injury on phosphorylated p38/JNK levels was attenuated by the pharmacological inhibition and genetic deletion of NK-1R *in vivo.* Furthermore, treatment with JNK or p38 inhibitors partially abolished apoptosis, G2/M arrest, and fibrogenesis mediated by SP in NK-1R-overexpressing HK-2 cells, and the combined use of JNK and p38 inhibitors enhanced these suppressive effects. Collectively, these data indicated that SP/NK-1R may induce injury and profibrogenic responses in HK-2 cells by activating JNK/p38 MAPKs.

In conclusion, we identify the SP/NK-1R axis as a novel pathway whose activation leads to renal inflammation and fibrosis. Our study uncovers a regulatory mechanism of NK-1R expression whereby TFAP4 increases NK-1R transcription by directly binding to its promoter. Moreover, SP/NK-1R axis activation, may stimulate p38/JNK signaling to promote cell apoptosis and G2/M arrest as well as proinflammatory/profibrogenic responses, resulting in renal fibrosis. Thus, targeting SP/NK-1R axis with NK-1R pharmacological antagonists may represent a promising treatment for renal inflammation and fibrosis in chronic and end-stage kidney disease.

## Data availability statement

The datasets presented in this study can be found in online repositories. The names of the repository/repositories and accession number(s) can be found below: GSE216743 (GEO) GSE66494 (GEO).

## Ethics statement

Human samples protocols gained approval from the Institutional Research Ethics Committee at the Seventh Affiliated Hospital, Sun Yat-sen University. The patients/participants provided their written informed consent to participate in this study. Animal experimental protocols gained approval from the Institutional Research Ethics Committee at the Sun Yat-sen University.

## Author contributions

EZ, YaL, and MZ were responsible for most of the experiments. XJ, recruited the patient samples and collected clinical data. YuL, NL and HG were responsible for statistical analysis and data interpretation. XS was responsible for the generation of schematic diagram. YX were responsible for modifying the manuscript. JL, H-YL and ZZ designed the study and prepared the manuscript. All authors contributed to the article and approved the submitted version. 
