# Supplementary material for: Targeting NK-1R attenuates renal fibrosis via modulating inflammatory responses and cell fate in chronic kidney disease
Source: Front Immunol. 2023 Mar 24;14:1142240. doi: 10.3389/fimmu.2023.1142240 (PMC10080018; doi:10.3389/fimmu.2023.1142240)
Supplement: Supplementary file 1 [file DataSheet_1.pdf]

Supporting information

Targeting NK-1R attenuates renal fibrosis via modulating inflammatory responses and cell fate in chronic kidney disease

Enyi Zhu[1*], Yang Liu[1*], Ming Zhong[1*], Yu Liu[1], Xi Jiang[3], Xiaorong Shu[4], Na Li[1], Hui Guan[1], Yin Xia[5], Jinhong Li[1#], Hui-yao Lan[2,6#], Zhihua Zheng[1#]

<u>Inventory of supplementary data</u>

1. Table S1..……………..……………….. Page 2

2. Table S2…………….………………….Page 3

3. Table S3…………….………………….Page 4

4. Table S4…………….………………….Page 5

5. Table S5…………….………………….Page 6

6. Supplementary Figure 1...………….….. Page 7

7. Supplementary Figure 2...……………...Page 8

8. Supplementary Figure 3...……………...Page 9

9. Supplementary Figure 4...……………...Page 10

10. Supplementary Figure 5...…………….Page 11

Table S1. The clinical characteristics of CKD patients.

Clinical variables	Control (n=6)	Patients with CKD (n=21)	*P*
Female/male (n)	1/5	5/16	*>0.999*
Age	45.67±8.31	42.81±11.13	*0.567*
White blood cell (10^9)	6.46±0.43	6.79±1.64	*0.413*
Hemoglobin (g/L)	141.83±18.19	114.43±19.48	*0.005**
Platelet (10^9)	258.5±76.24	270.86±88.06	*0.758*
Serum creatinine (Scr, µmol/L)	88.50±17.17	241.61±159.50	*0.0003****
Estimated glomerular filtrationrate (eGFR, µmol/L)	87.30±21.38	33.92±17.74	*0.0001*****
Blood urea nitrogen (BUN, mmol/L)	4.88±1.79	14.21±19.24	*0.0401**
Uric acid (UA, µmol/L)	347.33±94.01	482.21±129.10	*0.0695*
Albumin (g/L)	39.47±3.50	37.60±5.22	0.419
Kidney disease	Renal cancer (100%)	IgA nephrology (52.38%)<br>Diabetic nephropathy (28.57%)<br>Hypertension nephropathy (9.52%)<br>Lupus nephritis (4.76%)<br>Focal segmental glomerulosclerosis (4.76%)	

Table S2. The baseline features of CKD patients and control individuals.

Clinical variables	Control (n=20)	Patients with CKD (n=28)	*P*
Female/male (n)	3/17	8/20	0.319
Age	54.15±5.74	59.29±11.48	0.0723
White blood cell (10^9)	5.54±0.79	6.99±1.86	0.0007***
Hemoglobin (g/L)	146.80±14.26	107.96±27.53	0.0001****
Platelet (10^9)	233.20±42.79	214.07±62.17	0.241
Serum creatinine (Scr, µmol/L)	75.85±13.33	457.68±317.34	0.0001****
Estimated glomerular filtrationrate (eGFR, µmol/L)	94.89±9.25	21.24±18.06	0.0001****
Blood urea nitrogen (BUN, mmol/L)	4.83±1.08	18.97±12.09	0.0001****
Uric acid (UA, µmol/L)	348.16±47.32	431.97±114.37	0.0013**
Cystatin C (Cys C, mg/L)	0.83±0.14	3.37±1.62	0.0001****
Total cholesterol (TC, mmol/L)	4.47±0.58	4.51±1.53	0.9075
Triglyceride (TG, mmol/L)	1.10±0.35	1.25±0.59	0.2932
Low density lipoprotein cholesterol (LDL-C, mmol/L)	2.54±0.51	2.65±1.26	0.6875
High density lipoprotein cholesterol (HDL-C, mmol/L)	1.25±0.22	1.08±0.32	0.0472*
Albumin (g/L)	43.93±1.84	36.61±4.77	0.0001****

Table S3. Sequences of DNA oligonucleotides

Name	Sense Primer (5'-3')	Antisense Primer (5'-3')
**Primers for RT-qPCR**		
Human β-actin	AAGATGACCCAGATCATGTTTGAG	GCAGCTCGTAGCTCTTCTCCAG
Human TACR1	CTCAACCACAGAGACCATGC	GGGGGAGGAAGTAGATCAGC
Human TFAP4	GAGGGCTCTGTAGCCTTGC	GAATCCCGCGTTGATGCTCT
Human CTGF	CAGCATGGACGTTCGTCTG	AACCACGGTTTGGTCCTTGG
Human MMP9	ACGCAGACATCGTCATCCAGT	GGACCACAACTCGTCATCGTC
Mouse β-actin	CCCTGAAGTACCCCATTGAA	CTTTTCACGGTTGGCCTTAG
Mouse Tacr1	CTCCACCAACACTTCTGAGTC	TCACCACTGTATTGAATGCAGC
Mouse Tac1	AAGCGGGATGCTGATTCCTC	TCTTTCGTAGTTCTGCATTGCG
Mouse Collagen I	GCTCCTCTTAGGGGCCACT	CCACGTCTCACCATTGGGG
Mouse α-SMA	GTCCCAGACATCAGGGAGTAA	TCGGATACTTCAGCGTCAGGA
Mouse MCP-1	TTAAAAACCTGGATCGGAACCAA	GCATTAGCTTCAGATTTACGGGT
Mouse TNF-α	CATCTTCTCAAAATTCGAGTGACAA	TGGGAGTAGACAAGGTACAACCC
**Primers for ChIP**		
NK-1R	GCAAGTAGCAAGCAGCAAAA	TATAACCCCCTGCAGAGACG
Negative (GAPDH)	TACTAGCGGTTTTACGGGCGCACGT	TCGAACAGGAGGAGCAGAGAGCGAA
**Primers for cloning (Restriction enzyme sites were underlined; homologous arm was indicated by lowercase letters; initiation codon and stop codon were indicated by bold letters.)**		
**Cloning into pCDH**		
TACR1	agattctagagctagcgaattcGCCACC**ATG**GATAACGTCCTCCCGGT	atccttcgcggccgcggatcc**CTA**GGAGAGC ACATTGGAGGAG
TFAP4	agattctagagctagcgaattcGCCACC**ATG**GAGTATTTCATGGTGCCCAC	atccttcgcggccgcggatcc**TCA**GGGAAGCTCCCCGTCC
**Cloning into pGL3-basic**		
p-(−1.5/+0.1k)	cgagctcttacgcgtgctagcAAATCCCAGGCTCCGAAAAAATAC	acttagatcgcagatctcgagCTGGAGCTTCGTATCCAGCTG
p-mutA	TCCACTGGTATGCTTTCCCAAATCCTTCCTTCCT	GGGAAAGCATACCAGTGGAGGACAATCAACTTCC
p-mutB	AGGTATGCGCGCAGCTGCCGCGCTG	AGCTGCGCGCATACCTGACAACTGTCTCATTCCACC

Table S4. Comparison of clinical features between CKD patients with low and high renal NK-1R expression.

Clinical variables	Patients with NK-1R protein level		*P*
	Low expression (n=11)	High expression (n=10)	
Female/male (n)	6/5	5/5	>0.999
Age	47.74±8.80	41.80±13.69	0.703
White blood cell (10^9)	6.41±1.53	7.21±1.75	0.276
Hemoglobin (g/L)	123.36±14.62	104.60±20.01	0.023*
Platelet (10^9)	273.73±85.85	267.70±94.99	0.880
Serum creatinine (Scr, µmol/L)	176.80±78.73	312.90±197.14	0.065
Estimated glomerular filtrationrate (eGFR, µmol/L)	41.55±17.57	25.54±14.40	0.035*
Blood urea nitrogen (BUN, mmol/L)	8.51±2.77	20.47±27.03	0.197
Uric acid (UA, µmol/L)	522.75±133.60	437.63±133.97	0.135
Cystatin C (Cys C, mg/L)	1.93±0.49	3.20±1.69	0.043*
Total cholesterol (TC, mmol/L)	5.52±1.55	5.88±2.84	0.723
Triglyceride (TG, mmol/L)	1.87±0.88	2.33±1.21	0.328
Low density lipoprotein cholesterol (LDL-C, mmol/L)	3.03±0.92	3.27±2.16	0.745
High density lipoprotein cholesterol (HDL-C, mmol/L)	1.17±0.30	1.18±0.41	0.873
Albumin (g/L)	38.41±3.06	36.70±6.95	0.487
Urine protein (24h g/L)	2.16±1.51	5.54±3.91	0.317

Table S5. Comparison of clinical characteristics between CKD patients with low and high serum SP level.

Clinical variables	Patients with SP protein level		P
	Low expression (n=14)	High expression (n=14)	
**Female/male (n)**	**7/7**	**1/13**	**0.033***
**Age**	**63.00±11.58**	**55.57±10.48**	**0.087**
**White blood cell (10^9)**	**6.68±1.86**	**7.31±1.86**	**0.374**
**Hemoglobin (g/L)**	**114.86±26.87**	**101.07±27.38**	**0.190**
**Platelet (10^9)**	**221.71±48.18**	**206.43±74.7**	**0.526**
**Serum creatinine (Scr, µmol/L)**	**255.21±164.87**	**660.15±413.43**	**0.003****
**Estimated glomerularfiltrationrate (eGFR, µmol/L)**	**29.94±19.13**	**12.53±12.19**	**0.008****
**Blood urea nitrogen (BUN, mmol/L)**	**13.41±7.88**	**24.53±13.22**	**0.012***
**Uric acid (UA, µmol/L)**	**435.00±117.19**	**428.95±115.81**	**0.892**
**Cystatin C (Cys C, mg/L)**	**2.45±1.09**	**4.30±1.56**	**0.0011****
**Total cholesterol (TC, mmol/L)**	**4.83±1.60**	**4.18±1.44**	**0.265**
**Triglyceride (TG, mmol/L)**	**1.11±0.41**	**1.39±0.72**	**0.224**
**Low density lipoprotein cholesterol (LDL-C, mmol/L)**	**3.06±1.37**	**2.23±1.02**	**0.082**
**High density lipoprotein cholesterol (HDL-C, mmol/L)**	**1.16±0.38**	**1.01±0.24**	**0.223**
**Albumin (g/L)**	**37.69±5.41**	**35.53±3.94**	**0.238**

Supplementary Figure 1

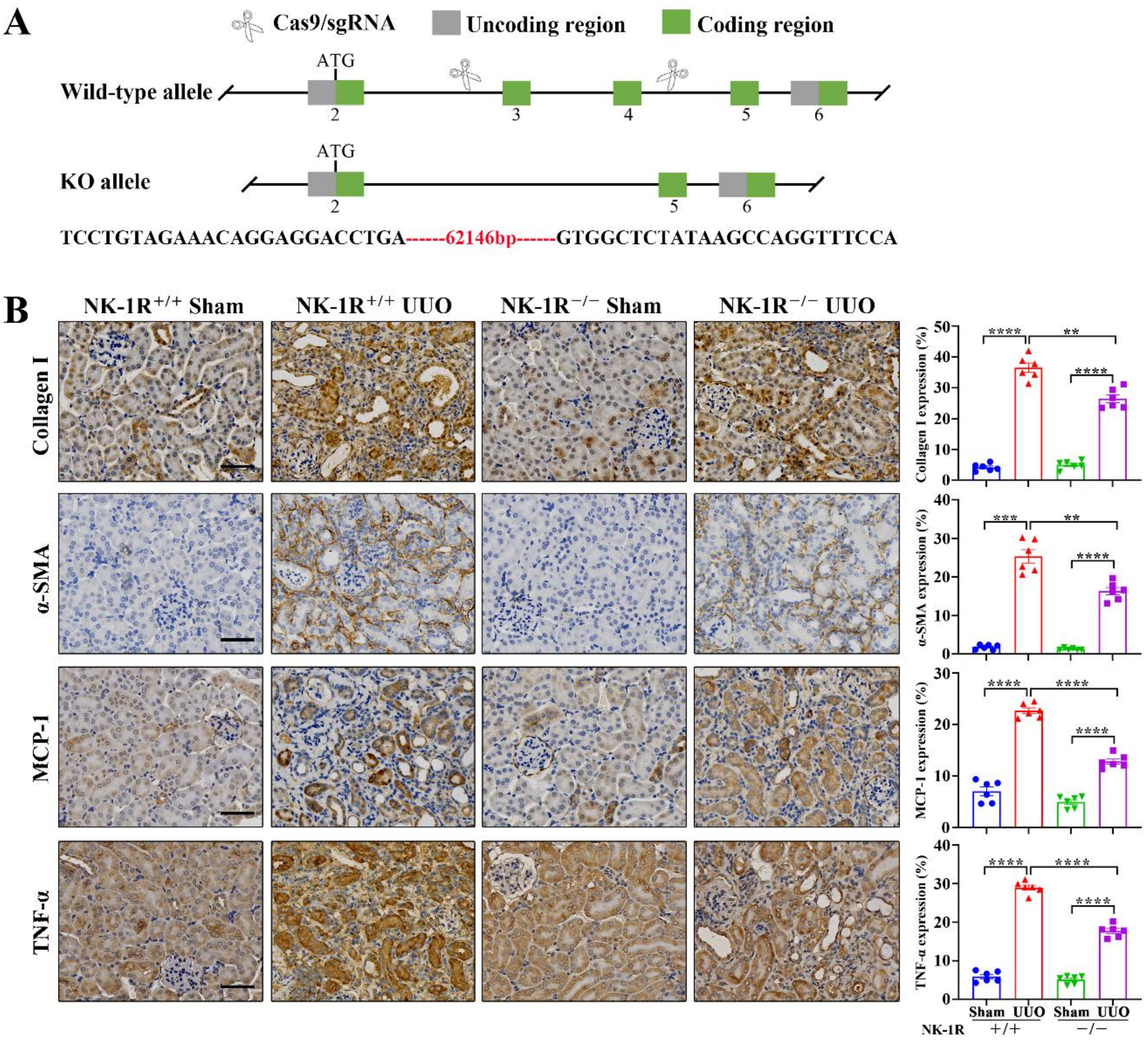

Supplementary Figure 1. Knockout of NK-1R attenuated renal inflammation and fibrosis in obstructed kidneys.

(A) The strategy for generating NK-1R knockout mice. (B) Immunochemistry staining displayed a reduction in Collagen I, α-SMA, MCP-1, and TNF-α protein levels following UUO. Scale bar, 50 μm. Data are shown as mean ± SEM from groups of six mice. **, $p < 0.01$; ***, $p < 0.001$; ****, $p < 0.0001$.

Supplementary Figure 2

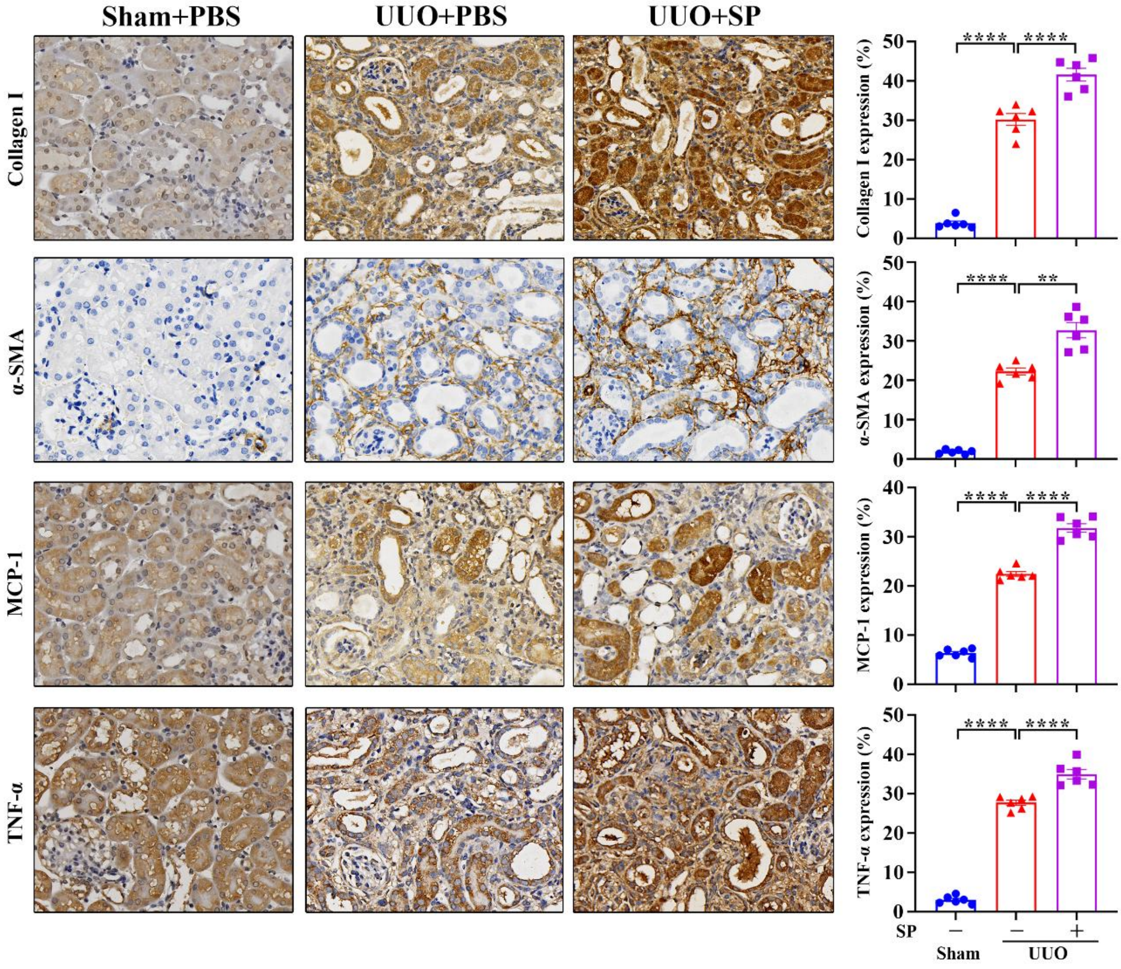

Supplementary Figure 2. SP administration aggravated renal inflammation and fibrosis upon UUO insult.

Immunochemistry staining showed that treatment with SP intensified the expression of Collagen I, α-SMA, MCP-1, and TNF-α upon UUO injury. Scale bar, 50 μm. Data are shown as mean ± SEM from groups of six mice. **, $p < 0.01$; ****, $p < 0.0001$.

Supplementary Figure 3

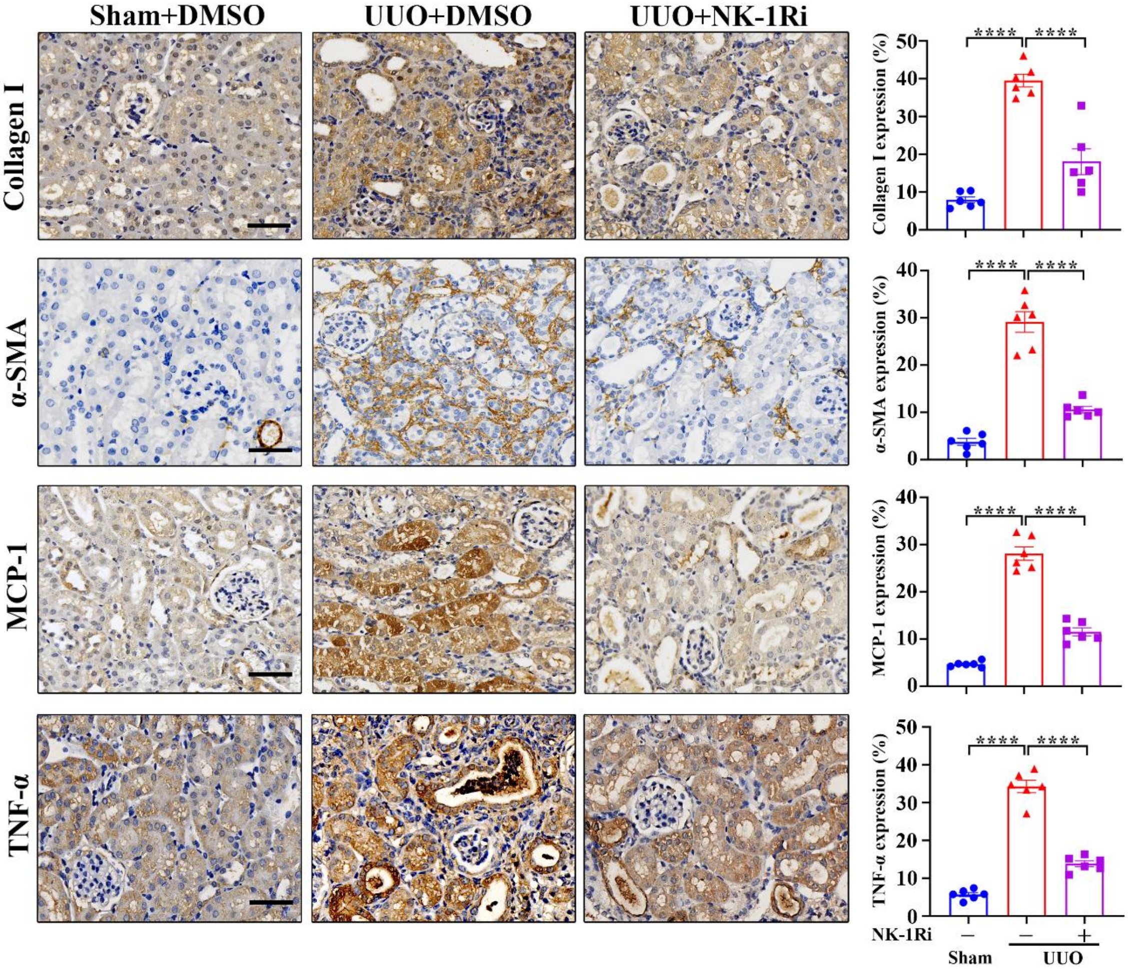

Supplementary Figure 3. Treatment with an NK-1R inhibitor alleviated UUO-stimulated renal inflammation and fibrosis in the kidney.

Immunochemistry staining showed that treatment with an NK-1R inhibitor attenuated the UUO-induced increase in Collagen I, α-SMA, MCP-1, and TNF-α levels. Representative images (left panels) and quantitative data (right panels) are shown. Scale bar, 50 µm. Data are shown as mean ± SEM from groups of six mice. ****, $p < 0.0001$.

Supplemeantary Figure 4

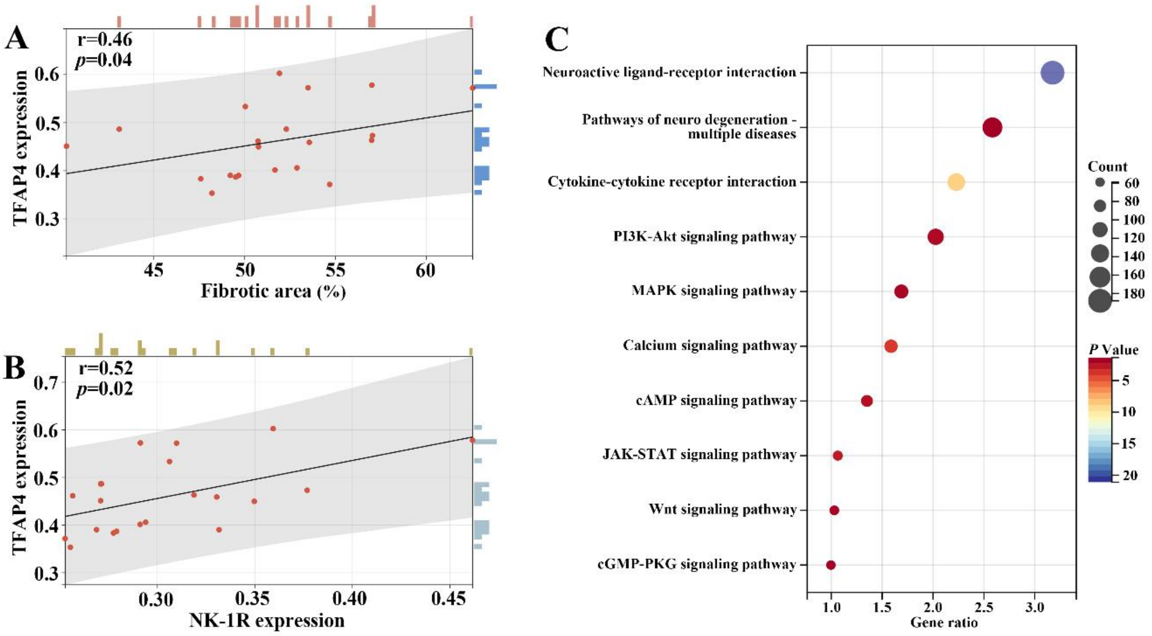

Supplementary Figure 4. TFAP4 expression was positively associated with fibrotic extent and NK-1R expression in CKD kidneys.

(A, B) Correlation between TFAP4 protein levels and fibrotic extent (A) and NK-1R protein levels (B) in 21 patients with CKD. (C) The KEGG enrichment of TFAP4 co-expressed genes in GSE66494. TFAP4 co-expressed genes were selected using Spearmen's correlation test in GSE66494 with a threshold of $p < 0.05$ and $|r| >= 0.3$.

Supplemeantary Figure 5

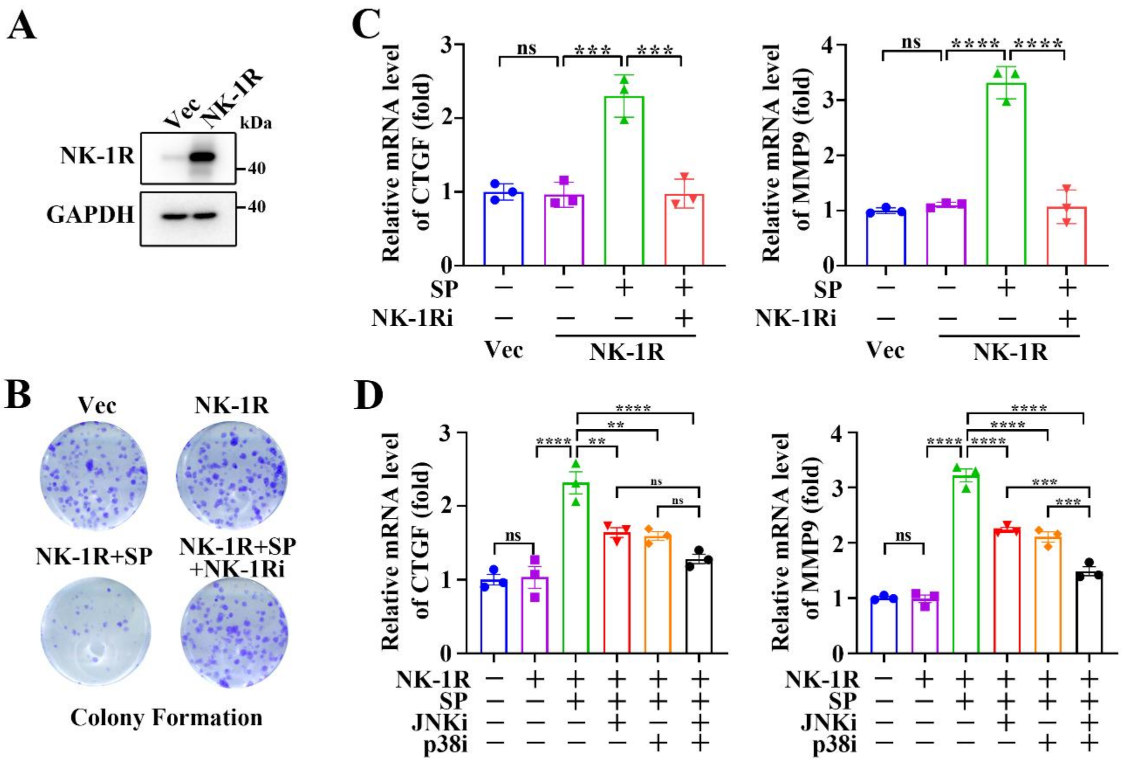

Supplementary Figure 5. The SP/NK-1R/MAPK pathway inhibited the growth of renal tubular epithelial cells and promoted fibrogenesis.

(A) Western blotting showed the overexpression of NK-1R in HK-2 cells. (B) The representative images of colony formation in NK-1R-overexpressed HK-2 cells. (C) RT-qPCR showed that treatment with an NK-1R inhibitor impeded the SP-induced elevation in the mRNA levels of profibrogenic genes in HK-2 cells stably overexpressing NK-1R. (D) RT-qPCR showed that p38 or JNK inhibitors or their combination decreased SP-stimulated increases in the mRNA levels of profibrogenic genes in NK-1R-overexpressed HK-2 cells. **, $p < 0.01$; ***, $p < 0.001$; ****, $p < 0.0001$; ns, not significant.
